# Supplementary material for: Endogenous stimuli-responsive separating microneedles to inhibit hypertrophic scar through remodeling the pathological microenvironment
Source: Nat Commun. 2024 Mar 6;15:2038. doi: 10.1038/s41467-024-46328-2 (PMC10917775; doi:10.1038/s41467-024-46328-2)
Supplement: Supplementary file 1 — Supplementary information [file 41467_2024_46328_MOESM1_ESM.pdf]

Supplementary Information

Endogenous Stimuli-Responsive Separating Microneedles to Inhibit Hypertrophic Scar through Remodeling the Pathological Microenvironment

Zhuo-Ran Yang[1#], Huinan Suo[2#], Jing-Wen Fan[3], Niannian Lv[1], Kehan Du[1], Teng Ma[1], Huimin Qin[1], Yan Li[2], Liu Yang[2], Nuoya Zhou[2], Hao Jiang[1], Juan Tao[2]* and Jintao Zhu[1]**

[1] Hubei Engineering Research Center for Biomaterials and Medical Protective Materials, School of Chemistry and Chemical Engineering, Huazhong University of Science and Technology (HUST), Wuhan 430074, China
[2] Department of Dermatology, Union Hospital, Tongji Medical College, HUST, Wuhan 430022, China
[3] Department of Radiology, Xijing Hospital, The Forth Military Medical University (FMMU), Xi'an, 710032, China
[#] These authors contributed equally to this work
E-mail: jtzhu@mail.hust.edu.cn (J. Z.); tjhappy@126.com (J. T.); hustjh@hust.edu.cn (H. J.)

Supplementary Methods

Materials. Polydimethylsiloxane (PDMS, Sylgard 184) was purchased from Dow Corning Corp. (Midland, Michigan, USA). Gelatin (from porcine skin), rhodamine B (RhB), hydrogen peroxide (H_2O_2) and methylacrylic acid were obtained from Sinopharm Chemical Reagent Co., Ltd. (Beijing, China). Methacrylic anhydride (MA) and deuterium oxide (D_2O) were obtained from Energy Chemical (Shanghai, China). 5-Fluorouracil (5-Fu) and ((1H-Benzo[d][1,2,3]triazol-1-yl)oxy)tri(pyrrolidin-1-yl)phosphonium hexafluorophosphate(V) (PyBOP) were purchased from Bide Pharmatech Co., Ltd. (Shanghai, China). Triisopropylsilane (TIS), trifluoroacetic acid (TFA), N,N-diisopropylethylamine (DIPEA), tetrakis (triphenylphosphine) palladium (0) ($Pd(PPh_3)_4$), phenylsilane ($PhSiH_3$), 2-hydroxy-1-(4-(2-hydroxyethoxy) phenyl)-2-methylpropan-1-one (Irgacure 2959) and bromoacetic acid were obtained from Aladdin (Shanghai, China). All amino acid derivatives involved in the synthesis were purchased from GL Biochem (Shanghai) Ltd. (Shanghai, China). Rink amide-MBHA resin and 2-CTC resin were obtained from Sunresin New Materials Co., Ltd (Xi'an, China). Dulbecco's modified Eagle medium (DMEM), penicillin-streptomycin solution, trypsin-EDTA solution (0.25% Trypsin, 0.02% EDTA), phosphate buffered solution (PBS), fetal bovine serum (FBS), paraformaldehyde and trypan blue staining solution (0.8%) were acquired from Biosharp Life Sciences (Hefei, China). Cell counting kit-8 (CCK-8), calcein/PI cell viability/cytotoxicity assay kit and reactive oxygen species (ROS) assay kit were obtained from Beyotime Biotechnology (Shanghai, China). Matrix metalloproteinase 9 (MMP9) was purchased from ACROBiosystems (Beijing, China) Anti-MMP2 and anti-MMP9 antibodies were purchased from Abcam (Cambridge, UK). 8-Hydroxy-2'-deoxyguanosine (8-OHdG) antibody was purchased from Santa Cruz Biotechnology (Dallas, USA). Other solvents were acquired from Beijing InnoChem Science & Technology Co., Ltd. (Beijing, China). All the chemicals were used as received without further purification. MNs master moulds were provided by Micropoint Technologies Pte. Ltd. (Singapore). Ultrapure water was obtained using an ultrapure water purifier system (GWB-1, PERSEE, China).

Synthesis and characterization of gelatin methacryloyl (GelMA). Briefly, 25 g of gelatin was dissolved in 250 mL of H_2O (60 °C), followed by stirring vigorously at 50 °C for 3 h and dropwise addition of 20 mL of MA. Afterwards, the reaction was quenched by adding 1 L of H_2O (50 °C). The mixed solution was stirred for another 15 min. Residual MA was removed by dialysis (8-14 KDa) at 50 °C for 7 days. After freeze-drying, GelMA was stored at 4 °C before use. GelMA was characterized by ^{1}H NMR. Gelatin and GelMA were dissolved in D_2O, respectively. ^{1}H NMR was obtained by a dual-channel fully digitized Fourier superconducting nuclear magnetic resonance spectrometer (AV400, BRUKER, Switzerland). The degree of methacryloylation was calculated by the following formula: DS (%) = 0.3836 mol × ($I_{5.7\ ppm}/I_{1.2\ ppm}$) × (100/0.0385 mol), where I represents the integral at the corresponding chemical shift[1,2].

Synthesis and characterization of 5-FuA-Pro-Pro. The procedure for synthesis of 5-FuA-Pro-Pro was similar to that of 5-FuA-Pep-MA. The difference was that synthesis of 5-FuA-Pro-Pro started from 2-chlorotrityl resin (0.55 mmol/g 1.0 equiv.). After 5-FuA coupling, 5-FuA-Pro-Pro was cleaved from the resin (1×20 min) with the addition of TFA/TIS/H_2O (1% : 2.5% : 2.5%). Post-procedure and characterization of the product were similar to those of 5-FuA-Pep-MA. ^{1}H NMR (400 MHz, D_2O): δ 7.64 (d, J = 5.8 Hz, 1H), 4.64 (d, J = 2.6 Hz, 1H), 4.62 (m, 1H), 4.48 (d, J = 17.0 Hz, 1H), 4.24 (dd, J = 8.7, 4.3 Hz, 1H), 3.65 (m, 1H), 3.59 (m, 1H), 3.56 (s, 1H), 3.52 (m, 1H), 2.23

(m, 1H), 2.13 (m, 1H), 1.96 (dd, J = 7.0, 5.3 Hz, 1H), 1.93 (m, 1H), 1.90 (s, 1H), 1.88 (d, J = 2.6 Hz, 2H), 1.86 (m, 2H). HRMS (ESI) m/z: $[M+Na]^+$ calcd for $C_{16}H_{19}FN_4NaO_6$, 405,1181; found, 405.1229.

Synthesis and characterization of FITC-Pep-MA. The synthesis procedure of FITC-Pep-MA was similar to that of 5-FuA-Pep-MA. The difference was that FITC was coupled to the N-terminal of the tetrapeptide (PPPK). Electrospray time-of-flight high-resolution mass spectrometry (ESI-HRMS) data were obtained on an Orbitrap mass spectrometer (Q Exactive, ThermoFisher Scientific, Germany). HRMS (ESI) m/z: $[M+H]^+$ calcd for $C_{52}H_{62}N_8O_{11}S$, 1007.4332; found, 1007.4554.

ROS-responsive cleavage of peptide prodrug. 300 mg of 5-FuA-Pep-MA was dissolved in PBS (10 mL) with different concentrations of H_2O_2. After different time of incubation at 37°C, the degradation degree of 5-FuA-Pep-MA was characterized by HPLC (UltiMate 3000, ThermoFisher Scientific, USA). The peak area obtained from the sample without H_2O_2 was recorded as A_0, while the peak area obtained from the sample with H_2O_2 was recorded as A_c. The cleavage rate was calculated by $[(A_0-A_c)/A_0] \times 100\%$ to evaluate the ROS-responsive cleavage of peptide prodrug. Each sample was measured in triplicate to calculate the mean and standard deviation.

Fabrication of MN patch moulds. The PDMS and Sylgard 184 were mixed (10:1). Then, the mixture was poured onto the surface of a pyramid-shaped MN positive mould and degassed using a vacuum defoaming device at ~0.08 MPa for 1 h at room temperature, followed by heating and curing at 80 °C for 5 h. After cooling down to room temperature, a PDMS MN negative mould was obtained after being demoulded, with a needle length of 650 μm, a needle distance of 500 μm and a needle number of 10 × 10 per patch. The PDMS negative moulds were used for MN fabrication.

Mechanical strength of MN patches. The mechanical properties of MN patches were tested using an *in-situ* uniaxial pulling pressurization system (IBTC-300SH, CARE Measurement & Control, China). Briefly, MNs were placed upwards on a stainless-steel plate, and compressed at a rate of 1 mm/min with a maximum loading force of 100 N. The correlation between the applied force and the displacement distance of MN patches with different crosslinking durations was recorded.

Swelling ability of crosslinked MN patches. The procedure was performed according to previous reports[1,3]. The swelling ratio of crosslinked MN patches was obtained by comparing the mass before and after adequate swelling. The dry weight (W_d) of each MN patch before swelling was recorded. MN patches were then inserted into a 1.4 wt.% agarose gel through a layer of parafilm. After adequate swelling for 24 h, the residual liquid on the patch surface was gently removed by filter paper and the wet weight (W_w) was recorded. The swelling rate was then calculated by $[(W_w-W_d)/W_d] \times 100\%$. Each sample was measured in triplicate to calculate the mean and standard deviation.

Drug loading capacity of MN tips. To evaluate the drug loading capacity of MN patches, GelMA-photoinitiator precursor solutions (0.2 g/mL of GelMA and 5 mg/mL of Irgacure 2959) with different concentrations of 5-FuA-Pep-MA were prepared. After MN patches were moulded without UV light crosslinking, MN tips and base layer of MN patches were separated with a small blade. Then, MN tips were dissolved in a certain volume of deionized water. Drug loading in MN patches was characterized by high performance liquid chromatography (HPLC; UltiMate 3000, ThermoFisher Scientific, USA) using the standard curve method. The values of drug loading were calculated as the content of 5-FuA. Each sample was measured in triplicate to calculate the mean and standard deviation.

Evaluation of crosslinking efficiency of MNs. The crosslinking efficiency of MNs was deduced by the degree of crosslinking reaction between 5-FuA-Pep-MA and GelMA. Specifically, 10 μL of

an equal concentration of 5-FuA-Pep-MA in GelMA-photoinitiator precursor solution (0.2 g/mL of GelMA and 5 mg/mL of Irgacure 2959) was exposed to UV light (365 nm, 400 mW/cm^2) for different durations. The precursor solution was subsequently diluted in an equal volume of PBS at 50 °C, followed by sufficient sonication for 30 min to dissolve free 5-FuA-Pep-MA. GelMA and the crosslinked 5-FuA-Pep-MA were filtered through a 0.45 μm membrane. The content of 5-FuA-Pep-MA at the free-form was characterized by HPLC (UltiMate 3000, ThermoFisher Scientific, USA). The peak area of the uncrosslinked sample is recorded as A_0, while the peak area of the crosslinked sample is recorded as A_t. The crosslinking efficiency is calculated by $[(A_0-A_t)/A_0] \times 100\%$. Each sample was measured in triplicate to calculate the mean and standard deviation.

Enzymatic degradation response of crosslinked MNs. The enzymatic degradation performance of MNs was assessed through in vitro degradation activity of crosslinked GelMA in the presence of MMP9. Specifically, 100 μL of GelMA solution (0.2 g/mL) with different crosslinking time was dried. After the crosslinked GelMA xerogel reached equilibrium swelling in PBS, the initial wet weight (W_0) was recorded, followed by incubation in PBS (containing MMP9, 30 U/mL) at 37 °C. The crosslinked GelMA was taken out from the solution at different time intervals, and the wet weight (W_t) was recorded after removal of the surface liquid. MMP9 was replenishable to the system every 12 h to ensure enzyme activity. The degradation of crosslinked GelMA was calculated by $[(W_0-W_t)/W_0] \times 100\%$. In addition, 100 μL of GelMA solution (0.2 g/mL) with crosslinking duration of 45 s was dried. After the crosslinked GelMA xerogel reached equilibrium swelling in PBS, the initial wet weight (W_0) was recorded by the same method, followed by incubation in PBS containing different concentrations of MMP9 (3 U/mL, 30 U/mL, 150 U/mL, 300 U/mL, respectively) at 37 °C. The crosslinked GelMA was taken out from the solution at different time intervals, and the wet weight (W_t) was recorded after removal of the surface liquid. The degradation of crosslinked GelMA was calculated by $[(W_0-W_t)/W_0] \times 100\%$. Each sample was measured in triplicate to calculate the mean and standard deviation. To visually confirm the enzymatic degradation of MNs, integral MN patches crosslinked for 45 s were inserted into an agarose gel (1.4 wt.%) containing MMP9 (300 U/mL) at 37 °C for 1 h. Then, the MN patches were taken out and freeze-dried for scanning electron microscope (SU8010, HITACHI, Japan).

Drug release profile of MN drug delivery platform. The drug release profile of crosslinked GelMA (0.2 g/mL) containing 5-FuA-Pep-MA (100 mM) was analyzed in PBS consisting of MMP9 (150 U/mL) and H_2O_2 (100 μM) at 37 °C. Specifically, the dried drug-loaded GelMA was dialyzed (1000 Da) in 5 mL of PBS containing MMP9 (150 U/mL) and H_2O_2 (100 μM). MMP9 was supplemented every 12 h to maintain enzyme activity. 50 μL of the solution was taken out at indicated time points, which was analyzed by HPLC (UltiMate 3000, ThermoFisher Scientific, USA) using the standard curve method. Each sample was measured in triplicate to calculate the mean and standard deviation. In addition, we also analyzed the drug release profile of crosslinked GelMA (0.2 g/mL) containing 5-Fu (100 mM) in PBS consisting of MMP9 (150 U/mL) and H_2O_2 (100 μM) at 37 °C using the same method.

In vitro effective transdermal depth of MNs. Fresh pig skin was obtained from supermarkets, then the oil stains were removed from surface for use. To evaluate the effective transdermal depth of MNs and the distribution of drugs at different skin depths, MN patch loaded with RhB was vertically pressed into fresh pig skin for 5 min. The MN patch was carefully removed and the pig skin was subjected to confocal laser scanning microscope (CLSM; FV1200, Olympus, Japan). After determining the *xy*-plane with the highest and lowest fluorescence intensity in the CLSM,

fluorescence images were obtained from the *xy*-plane with a scanning interval of 20 μm along the *z*-axis to display the diffusion of the dye in the skin.

Ex vivo penetration capacity of MNs in hypertrophic scar (HS) tissue. The HS model was established according to the method described in this study, and a hard and dense HS was formed in the wound with a raised and inflamed skin surface. HS tissues were removed by surgery after general anesthesia. To assess the penetration capacity of MN patches in HS tissues, MN patches were pressed firmly on the surface of HS for 5 min. After removement of MN patches, the formed microholes were stained with trypan blue (a kind of tissue labeling dye) solution for 5 min and washed with saline. Then, the HS tissues were photographed with a digital camera (HUAWEI, China). Besides, HS tissue sections were stained with hematoxylin and eosin (H&E), and subjected to a biological inverted microscope (IX71, Olympus, Japan).

In vivo subcutaneous retention time of separating MN patches. To verify the subcutaneous retention ability of the separating MNs, MN Patch was pressed into the clipped skin in the middle of the ventral surface of rabbit ear. 5 min later, the MN tips were separated from the base layer by slight force along the radial direction of MN patch. Photographs were taken with a digital camera (HUAWEI, China) at different time intervals to display the subcutaneous retention of MN tips. At the same time, integral MNs were also operated as control. In addition, separating and integral MN patches loaded with FITC-Pep-MA separately pierced the skin of mice and imaged using an in vivo imaging system (In-Vivo FX PRO, BRUKER, USA) for small animal imaging. The fluorescence images were also captured in histological sections to visually display the subcutaneous retention of MN tips.

In vitro cell viability and cytotoxicity. The CCK-8 assays were used to detect the cytotoxicity of 5-Fu and 5-Fu derivatives towards NIH/3T3 fibroblasts. Briefly, NIH/3T3 fibroblasts were cultured with DMEM medium (containing 10% FBS and 1% penicillin-streptomycin) at 37 °C with 5% CO_2. Then, NIH/3T3 fibroblasts were seeded in 96-well plates at a density of 5×10^3 cells/well and cultured for 12 h to adhere to the bottom of the plates. Subsequently, the medium was replaced with 100 μL of fresh medium containing 5-Fu, 5-FuA, 5-FuA-Pep-MA and 5-FuA-Pro-Pro at concentrations of 80, 125, 250 μM, respectively. The cells treated with the neat medium were taken as the control group. After 48 h of incubation, the CCK-8 kit was added, and the absorption of each well at 450 nm was measured with a microplate reader (Multiskan MK3, ThermoFisher Scientific, USA) to calculate the cell viability.

Live/dead staining assay. Live/dead staining assays were implemented to visualize the toxicity of the drugs (5-Fu, 5-FuA, 5-FuA-Pep-MA and 5-FuA-Pro-Pro). Briefly, NIH/3T3 fibroblasts were cultured in 96-well plates for 12 h. Then, NIH/3T3 fibroblasts were incubated with 100 μL of fresh medium containing 5-Fu, 5-FuA, 5-FuA-Pep-MA and 5-FuA-Pro-Pro at concentrations of 80 and 250 μM for 48 h, respectively. Then, 100 μL of staining solution was added to each well and incubated for another 30 min. After incubation, 96-well plates were imaged with an inverted fluorescence microscope (IX71, Olympus, Japan).

In vitro wound scratch assay. Wound scratch assays were used to evaluate the migration of NIH/3T3 fibroblasts in the presence of 5-FuA, 5-FuA-Pep-MA and 5-FuA-Pro-Pro, respectively. NIH/3T3 fibroblasts were seeded into 24-well plates at a density of 2×10^4 cells/well for 8 h. Then, the sterile pipette tips were used to draw the scratch in the center of the wells, and the cells scratched off were washed away with PBS for 3 times. 500 μL of drug solutions consisting of 80 μM of 5-FuA (5-FuA-Pep-MA or 5-FuA-Pro-Pro) and 0.5% FBS were added into the wells, respectively.

The migration of cells was imaged with an inversion microscope at 0, 24 and 48 h. The area of the scratch was analyzed by Image J, and the wound healing rate (WHR) was calculated according to the following equation: WHR (%) = $(A_0 - A_t)/A_0 \times 100\%$, where A_0 and A_t represented the area of scratch at 0 h and t h, respectively.

Intracellular ROS scavenging. ROS assay kit was used to observe the elimination of intracellular ROS by 5-FuA-Pep-MA. Specifically, HSFbs were seeded in 8-well confocal dishes at a density of 1×10^4 cells/well and cultured for 12 h to adhere to the bottom of the dishes. Then, adding ROSup (positive control in the kit) stimulated cells to produce endogenous ROS, and 5-FuA-Pep-MA of 50, 100 and 200 μM were added to incubate the cells for 12 h. The medium was replaced with 2,7 dichlorofluorescein diacetate (DCFH-DA) and incubated for 20 min. Untreated cells were used as the control group. After incubation, 8-well confocal dishes were imaged with a CLSM (FV1200, Olympus, Japan).

Immunohistochemical, fluorescence and immunofluorescence staining of HS tissues. The ROS levels in HS tissues were measured by the fluorescence and immunofluorescence methods on day 43rd. Dihydroethidium (DHE) staining was performed to assess the ROS levels in the pathological microenvironment of HS with different treatments. Meanwhile, 8-OHdG staining was performed to assess the oxidative damage caused by ROS. The expression of MMP2 and MMP9 in HS tissues were measured by immunohistochemistry and immunofluorescence methods on day 43rd. Briefly, the tissue sections were deparaffinized, rehydrated, incubated in an antigen retrieval solution and blocking serum, and stained with primary antibodies to MMP2 (1:100) and MMP9 (1:100). For immunohistochemistry staining, the tissue sections were incubated with the secondary antibody, followed by the addition of DAB enzyme substrate to react with the tissue sections. Subsequently, H&E staining was used to counterstain the tissue sections. For immunofluorescence staining, Alexa Fluor 594-conjugated donkey anti-mouse IgG(H+L) (1:400) secondary antibodies were added to the tissue sections. Nuclear staining was performed with 4, 6-diamidino-2-phenylindole (DAPI). The irrelevant isotype-matched antibodies were used as negative controls. The images of the stained sections were captured by panoramic digital slide scanners (3D HISTECH, Hungary).

RNA sequencing and sample collection. Transcriptome studies on gene function and gene structure from an overall level reveal specific biological process and molecular mechanism in the process of disease occurrence, which have been widely used in basic scientific research, clinical diagnosis and medical research. Here, RNA sequencing (RNA-seq) technology was applied to investigate the mechanism of drug-loaded MNs on HS treatment. On the 43rd day (one week after the 3rd administration), the full thickness scar tissues of rabbit ears from groups 1, 2, 4 and 5 were harvested to perform bulk RNA-seq analysis. Total RNA was used as input material for the RNA sample preparations. Briefly, mRNA was purified from total RNA using poly-T oligo-attached magnetic beads. RNA integrity was assessed using the RNA Nano 6000 Assay Kit of the Bioanalyzer 2100 system (Agilent Technologies, CA, USA). PCR products were purified (AMPure XP system) and library quality was assessed on the Agilent Bioanalyzer 2100 system. High throughput sequencing was completed with an Illumina NovaSeq 6000 System (Illumina Inc., San Diego, CA).

Quality control. Firstly, raw data (raw reads) of fastq format were processed through in-house perl scripts. Then, clean data (clean reads) were obtained by removing reads containing adapter, reads containing ploy-N and low quality reads from raw data. Simultaneously, Q20, Q30 and GC content

and the clean data were calculated. The following gene analyses were based on the clean data with high quality.

Differential expression analysis. Differential expression analysis was performed using the DESeq2 R package. DESeq2 estimate variance-mean dependence in count data from high-throughput sequencing assays and determine differential expression based on the negative binomial distribution. The resulting *P* values were adjusted using the Benjamini and Hochberg's approach for controlling the false discovery rate (FDR). Differentially expressed genes (DEGs) were set with the criteria of absolute value of fold change > 2 (|log2FC| > 1) and a false discovery rate (FDR) < 0.05.

GO and KEGG enrichment analysis of differentially expressed genes. Functions of DEGs were analyzed using gene ontology (GO) and Kyoto encyclopedia of genes and genomes (KEGG) pathway analyses. The GO analyses consisted of biological process (BP), molecular function (MF), and cellular component (CC). GO terms with a *P* value < 0.05 were considered significantly enriched.

Gene set enrichment analysis. Gene set enrichment analysis (GSEA) is a computational approach that uses predefined gene sets to identify significant biological changes between two biological states. The genes were ranked according to the degree of differential expression in the two samples, and then the predefined gene set were tested to see if they were enriched at the top or bottom of the list. GSEA is especially useful when gene expression changes are minimal or moderate. We used the local version of the GSEA analysis tool (version 4.1.1, Broad Institute, Massachusetts Institute of Technology, USA).

Protein-protein interaction network analysis and hub genes. Protein-protein interactions (PPI) analysis of DEGs was based on the STRING database (https://cn.string-db.org/), and the confidence score ≥ 0.4 was used as the threshold. The PPI network was visualized by using Cytoscape software. We selected the hub genes (highly connected genes) using MCODE and cytoHubba plugin of Cytoscape software.

Single cell RNA sequencing and tissue dissociation. On the 43rd day (one week after the 3rd administration), 2 rabbit ear scar tissues without treatment (group 2) and 2 rabbit ear scar tissues with drug-loaded MNs treatment (group 4) were collected and performed scRNA-seq. The rabbit ear scar tissues included 5 layers, top to bottom: ventral epidermis, ventral dermis, cartilage, dorsal dermis and dorsal epidermis. The ventral epidermis and dermis have the characteristic features of HS. After washing two times in PBS, the ventral epidermis and dermis of the rabbit ear scar tissues were saved and cut into small pieces to further disassociated in the tissue dissociation solution. Single cell suspension was obtained by filtration through a 40 μm filter. After the samples were centrifuged, the supernatant discarded and the sediment was resuspended. Red blood cells were removed with lysis buffer. Cell viability were detected by trypan blue staining. If the cell viability is less than 80%, live cells would be further purified by a Dead Cell Removal Kit.

Single-cell library preparation and sequencing. Single-cell gel beads-in-emulsion generation, barcoding, sample cleanup, cDNA amplification, and cDNA library construction were performed. The raw FASTQ sequence reads were preprocessed by Cell Ranger software. Potential technical bias to further correct batch effect across different samples were removed. Unique molecular identifier (UMI) counting was identified by cellranger count for each sample, and then processed by R package Seurat for downstream analysis.

Dimensional reduction and clustering. Cells were displayed on the unsupervised uniform manifold approximation and projection (UMAP) plot. Cell clusters were annotated as cell types

according to the expressions of the identified differential genes and typical cellular markers reported in the previous literature.

Cell-cell interaction network analysis. To analyze cell-cell communication based on single-cell transcriptomic datasets, we used the R package CellChat to identify the potential interactions between different cell types. The CellChatDB database contains a curated repository of ligand-receptor interactions and a statistical framework for inferring lineage-specific interactions, which was imported to construct the ligand–receptor interactions.

Supplementary Figures

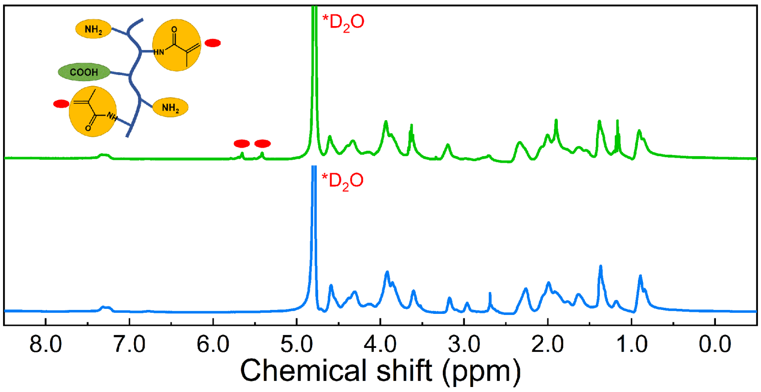

Supplementary Fig. 1 Characterization of GelMA. ^{1}H NMR spectra of GelMA in D_2O. (DS (%) = 0.3836 mol × ($I_{5.7\ ppm}/I_{1.2\ ppm}$) × (100/0.0385 mol) = 73.3%)

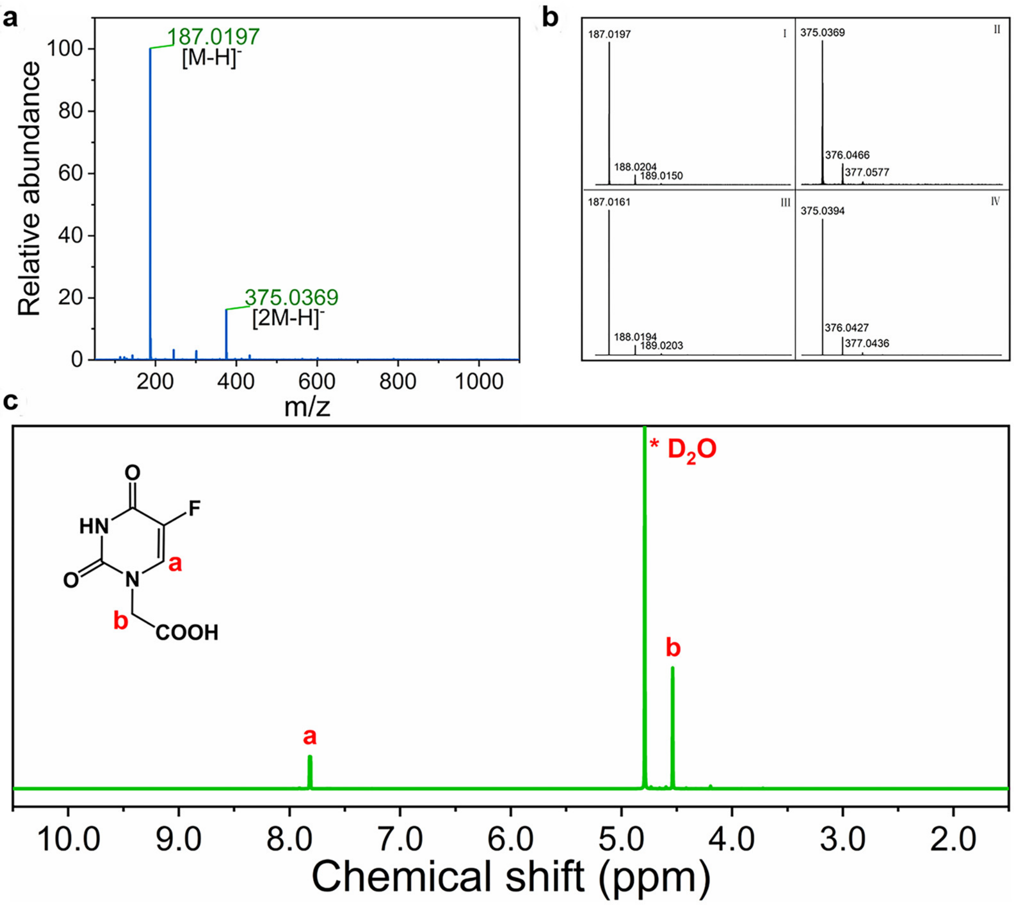

Supplementary Fig. 2 Characterization of 5-FuA. a, ESI-HRMS (negative) spectrum of 5-FuA. **b,** The peaks of isotopic distribution corresponding to the peaks at m/z = 187.0197 ([M-H]$^-$) and 375.0369 ([2M-H]$^-$), experimental (I and II) and simulative (III and IV, calculations for $C_6H_4FN_2O_4$ and $C_{12}H_9F_2N_4O_8$, respectively). **c,** ^{1}H NMR spectrum of 5-FuA in D_2O. The inset is the structure of 5-FuA.

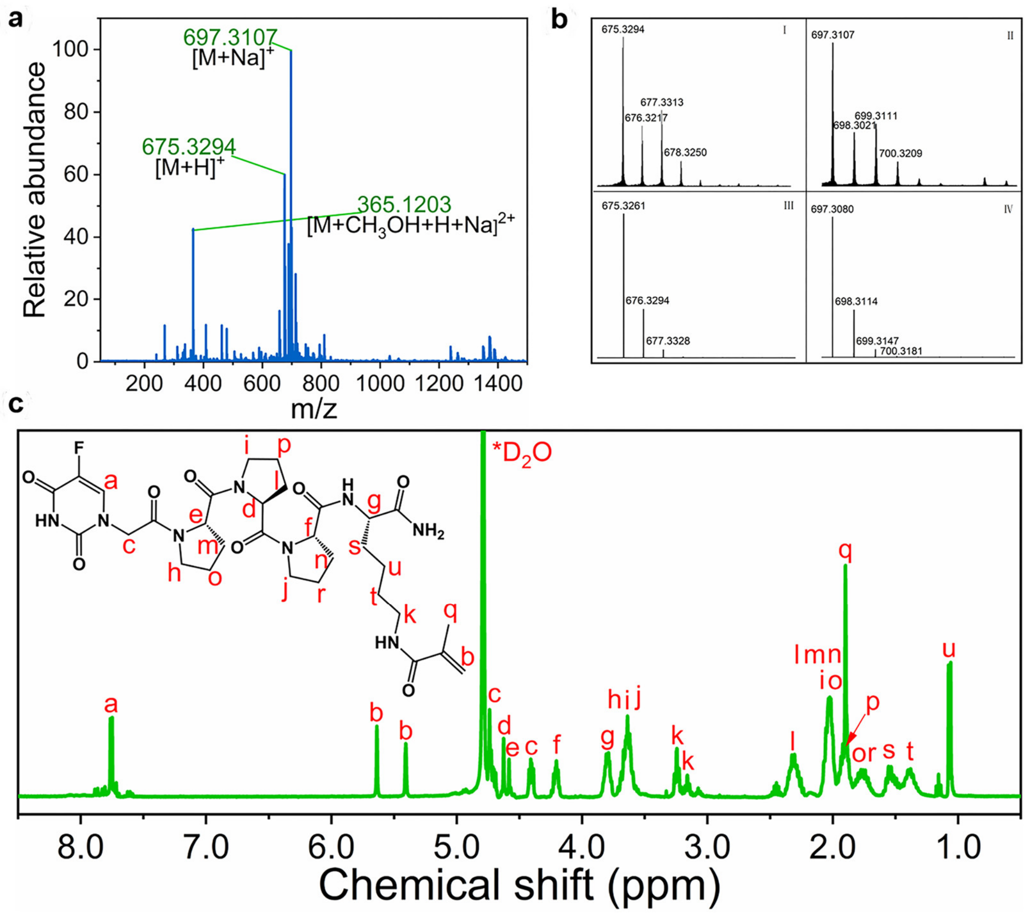

Supplementary Fig. 3 Characterization of 5-FuA-Pep-MA. a, ESI-HRMS (positive) spectrum of 5-FuA-Pep-MA. **b,** The peaks of isotopic distribution corresponding to the peaks at m/z = 675.3294 ($[M+H]^+$) and 697.3107 ($[M+Na]^+$), experimental (I and II) and simulative (III and IV, calculations for $C_{31}H_{44}FN_8O_8$ and $C_{31}H_{43}FN_8NaO_8$, respectively). **c,** ^{1}H NMR spectrum of 5-FuA-Pep-MA in D_2O. The inset is the sequence of 5-FuA-Pep-MA.

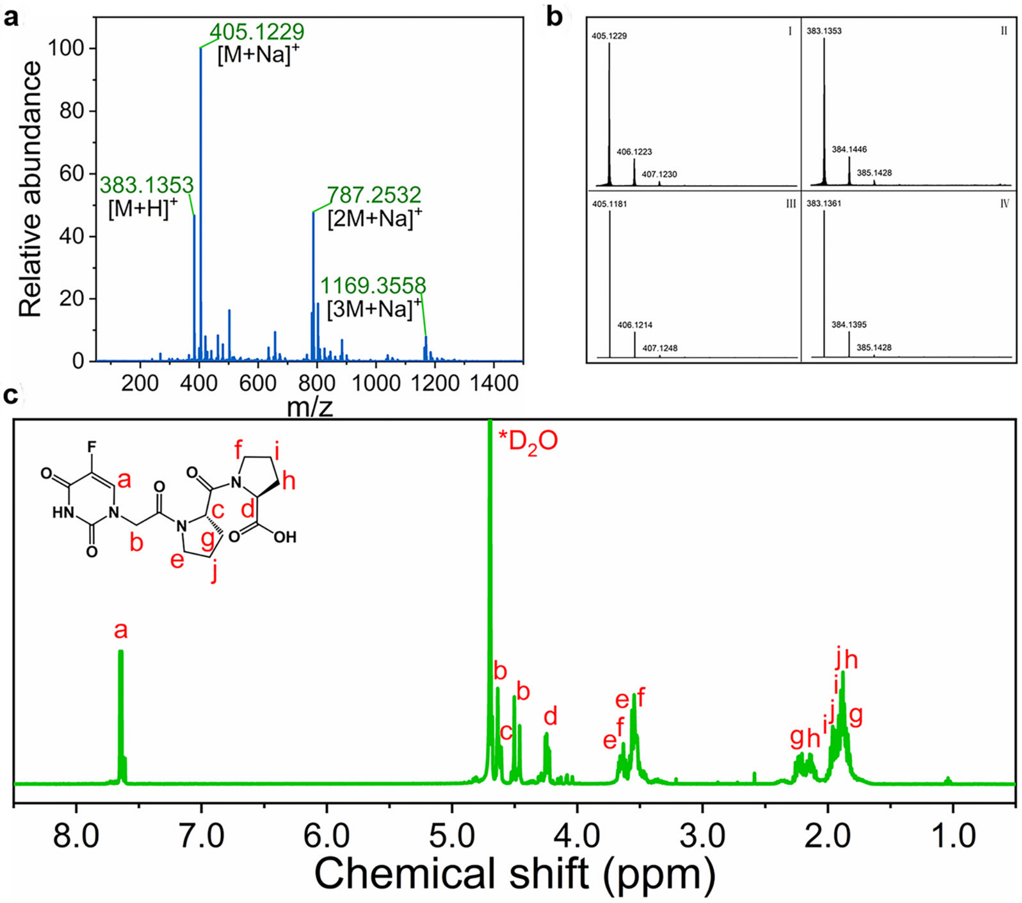

Supplementary Fig. 4 Characterization of 5-FuA-Pro-Pro. a, ESI-HRMS (positive) spectrum of 5-FuA-Pro-Pro. **b,** The peaks of isotopic distribution corresponding to the peaks at m/z = 405.1229 ($[M+Na]^+$) and 383.1353 ($[M+H]^+$), experimental (I and II) and simulative (III and IV, calculations for $C_{16}H_{19}FN_4NaO_6$ and $C_{16}H_{20}FN_4O_6$, respectively). **c,** ^1H NMR spectrum of 5-FuA-Pro-Pro in D_2O. The inset is the sequence of 5-FuA-Pro-Pro.

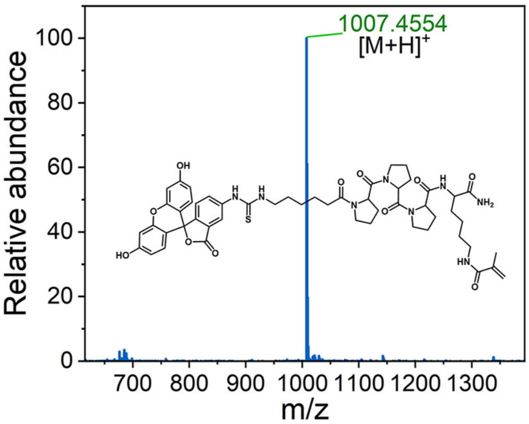

Supplementary Fig. 5 Characterization of FITC-Pep-MA. ESI-HRMS (positive) spectrum of FITC-Pep-MA. The experimental peak at m/z = 1007.4554 ($[M+H]^+$) corresponding to the simulative peak at m/z = 1007.4332 (calculations for $C_{52}H_{62}N_8O_{11}S$). The inset is the sequence of FITC-Pep-MA.

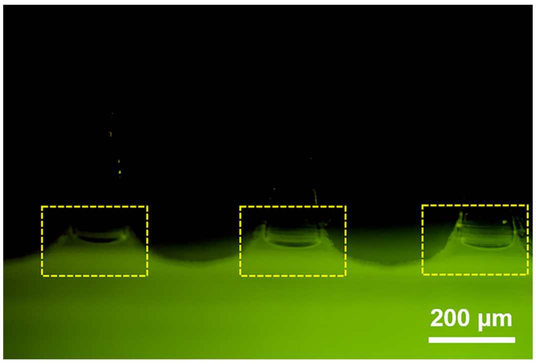

Supplementary Fig. 6 Characterization of MNs patches. Fluorescence image of MN patch with FITC-labeled gelatin as base layer. Representative images from n = 3 independent experiments.

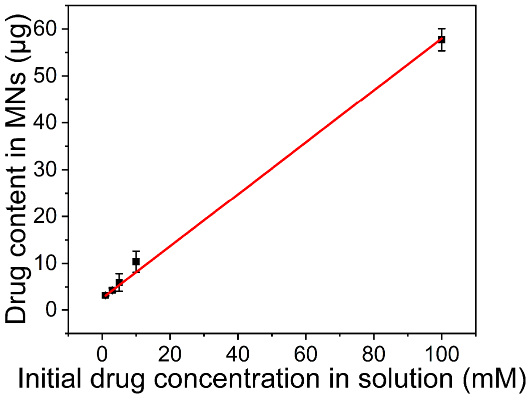

Supplementary Fig. 7 Drug loading capacity of MN patches. Relationship between the drug content in the needles of the patch and the initial drug concentration in solution used to prepare the MNs. The data are presented as mean ± SD (n = 3 independent experiments).

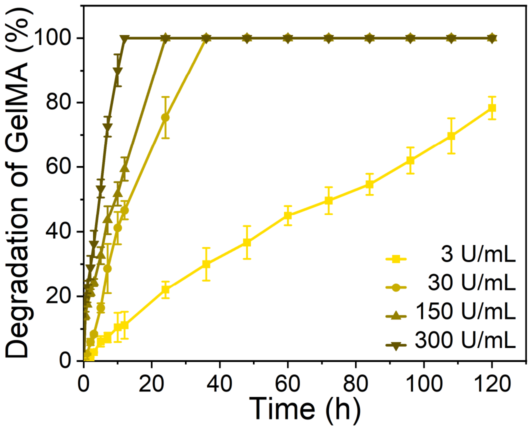

Supplementary Fig. 8 Enzymatic degradation response of crosslinked GelMA. Effects of different enzyme activities on degradation rates. The data are presented as mean ± SD (n = 3 independent experiments).

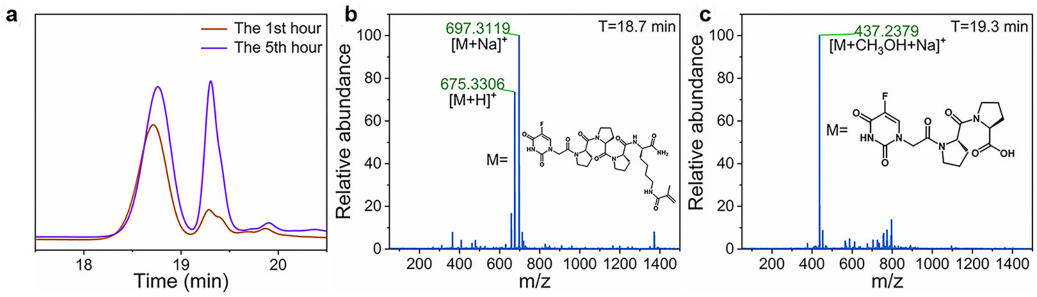

Supplementary Fig. 9 Released fragments from MNs drug delivery platform. a, HPLC for drug release at different time points. **b,** ESI-HRMS (positive) spectrum of the effluent of HPLC at 18.7 min. **c,** ESI-HRMS (positive) spectrum of the effluent of the HPLC at 19.3 min.

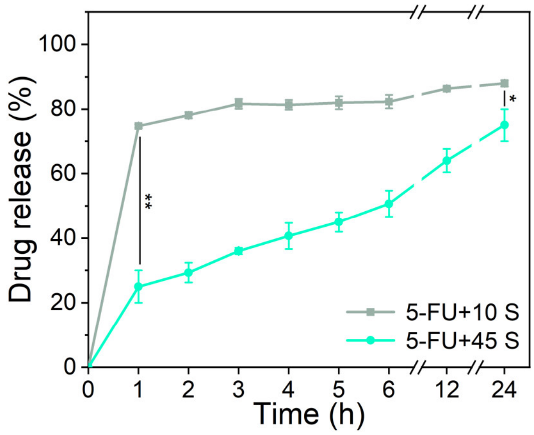

Supplementary Fig. 10 Drug release profile of MNs drug delivery platform. Drug release curves of single corresponding MNs drug delivery platform with different crosslinking durations. The data are presented as mean ± SD (n = 3 independent experiments). The data were analyzed by t test. $*P < 0.05$, $**P < 0.01$.

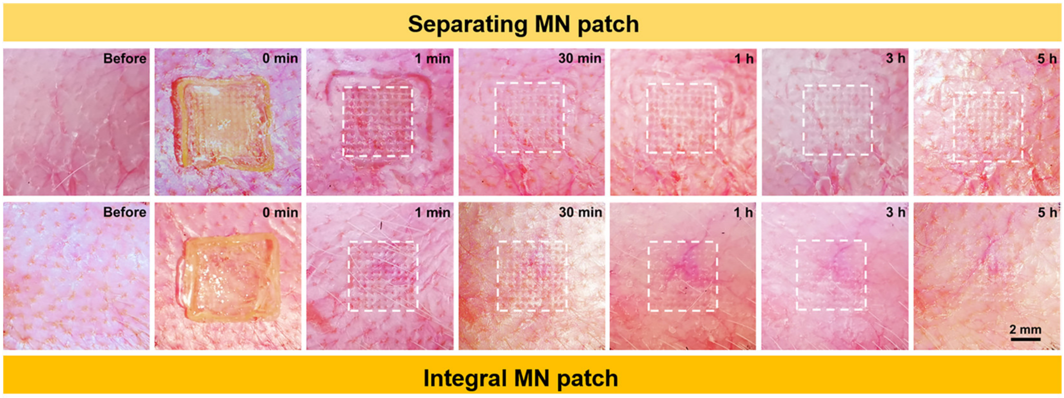

Supplementary Fig. 11 In vivo subcutaneous retention time of separable MN patches. Photographs of rabbit ear skin tissues treated with separating MN patch (top) and integral MN patch (bottom). The scale bar in the last image can be applied to the others. Representative images from n = 3 independent experiments.

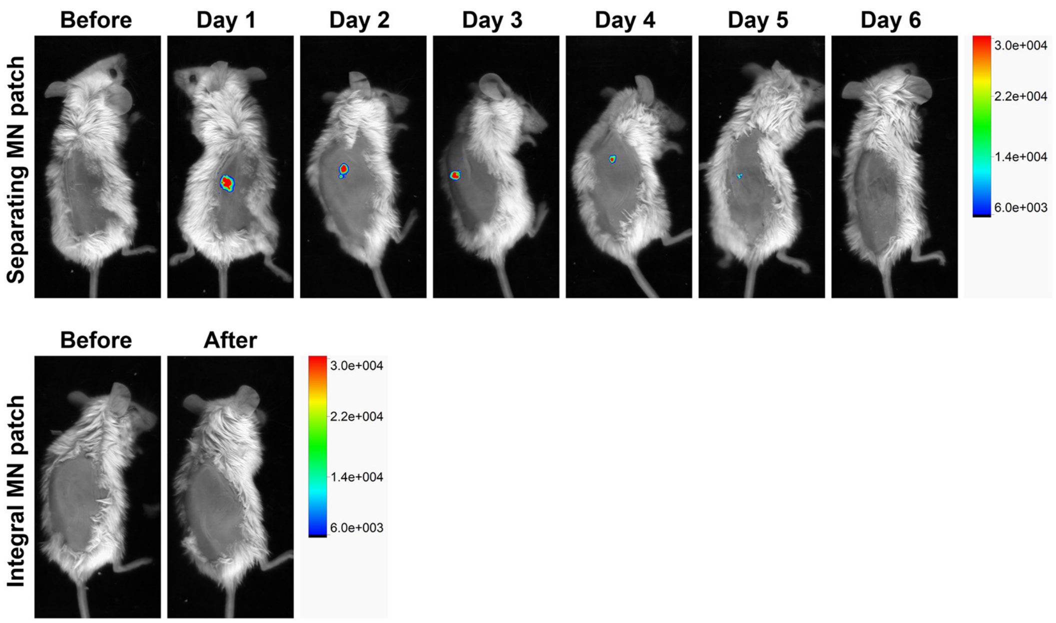

Supplementary Fig. 12 Subcutaneous retention of separating MN patches. Representative in vivo fluorescent images of mice at different time points treated with separating MN patch or integral MN patch. Representative images from n = 5 independent experiments.

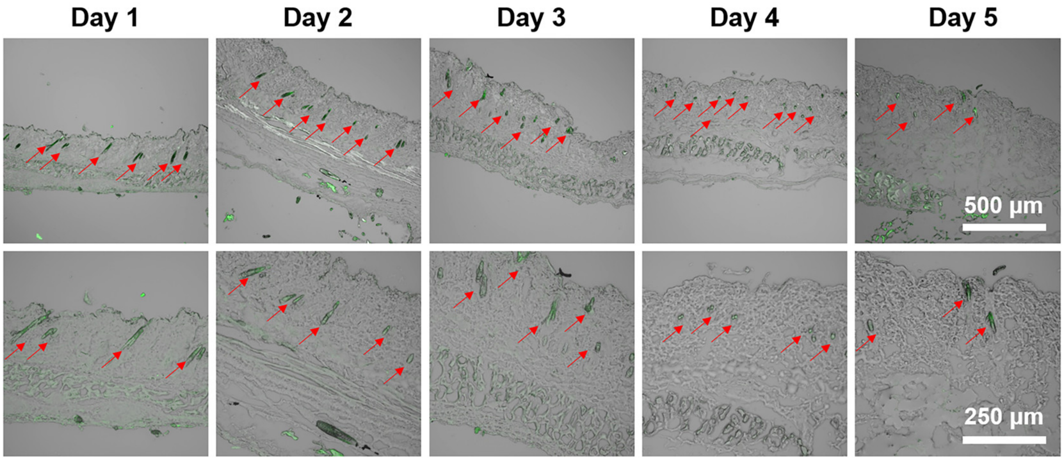

Supplementary Fig. 13 Subcutaneous retention of separating MN patches. Merged microscopy images of histological sections at different time points treated with separating MN patch. The scale bar in the last image can be applied to the others. Representative images from n = 3 biologically independent samples.

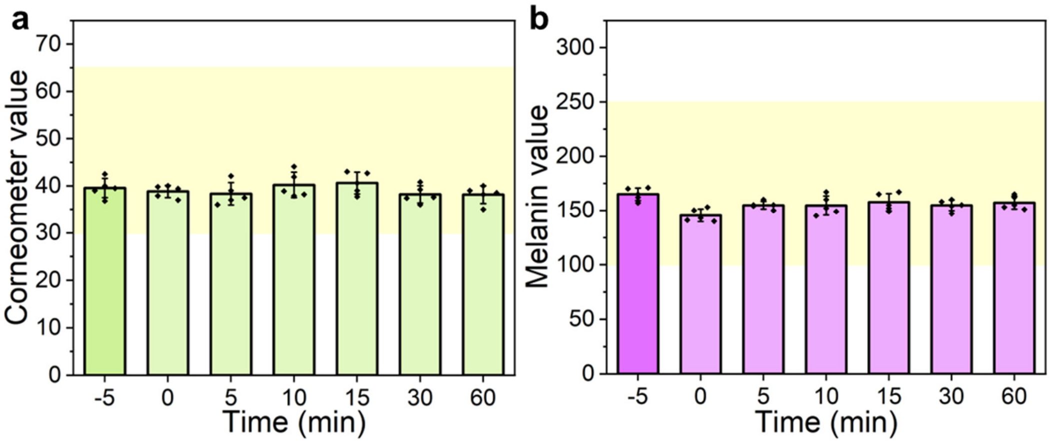

Supplementary Fig. 14 Acute skin irritation test of separating MN patches. a, Corneometer value from the treated skin of 5 healthy volunteers prior and after the separating MN patch. **b,** Melanin value prior and after the separable MN patch. The yellow ranges represent the normal range. The data are presented as mean ± SD (n = 5 independent experiments).

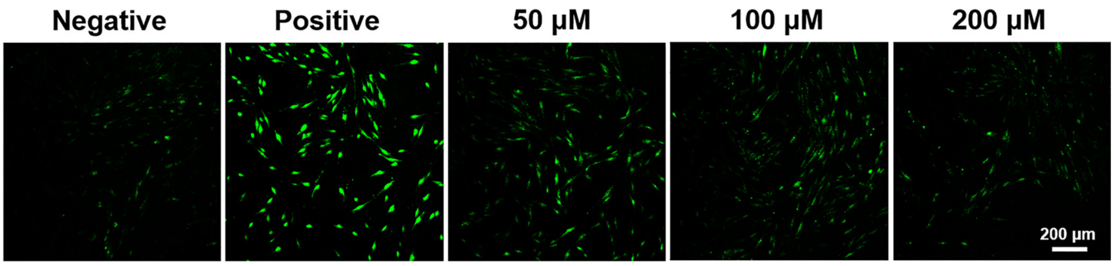

Supplementary Fig. 15 Intracellular ROS scavenging of the HSFb. Intracellular ROS levels of the HSFb treated with different concentrations of 5-FuA-Pep-MA. The scale bar in the last image can be applied to the others. Representative images from n = 3 independent experiments.

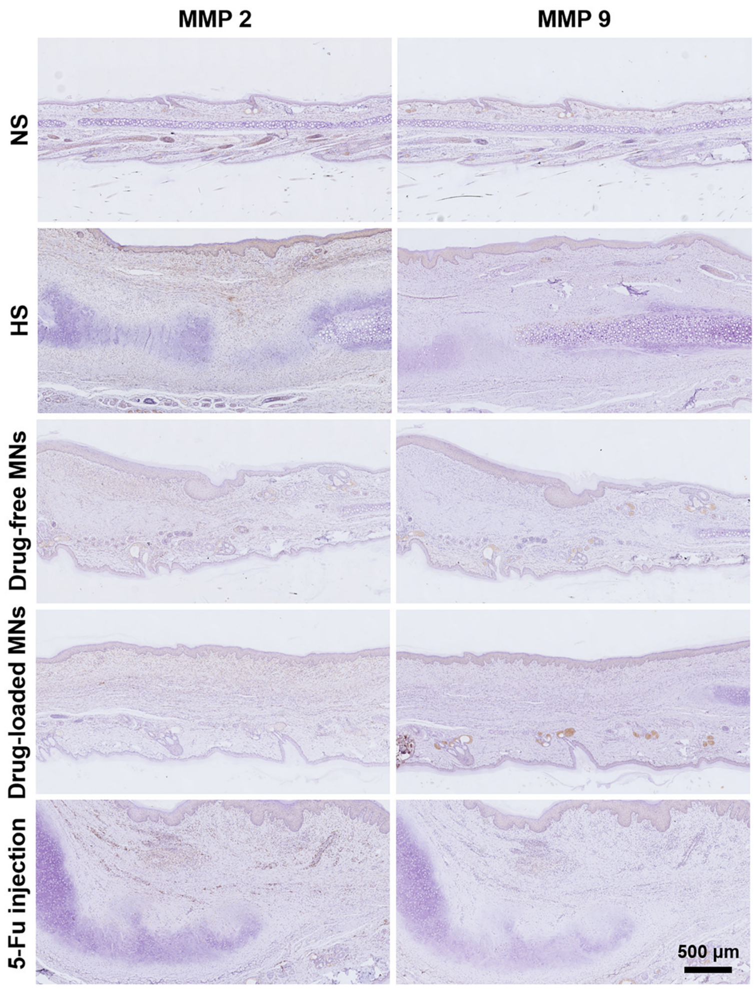

Supplementary Fig. 16 The expression of MMP2 and MMP9 in the microenvironment of NS and HS under different treatments. The immunohistochemistry staining for MMP2 (first column) and MMP9 (second column) in NS and HS tissues under different treatments. The scale bar in the last image can be applied to the others. Representative images from n = 3 biologically independent samples.

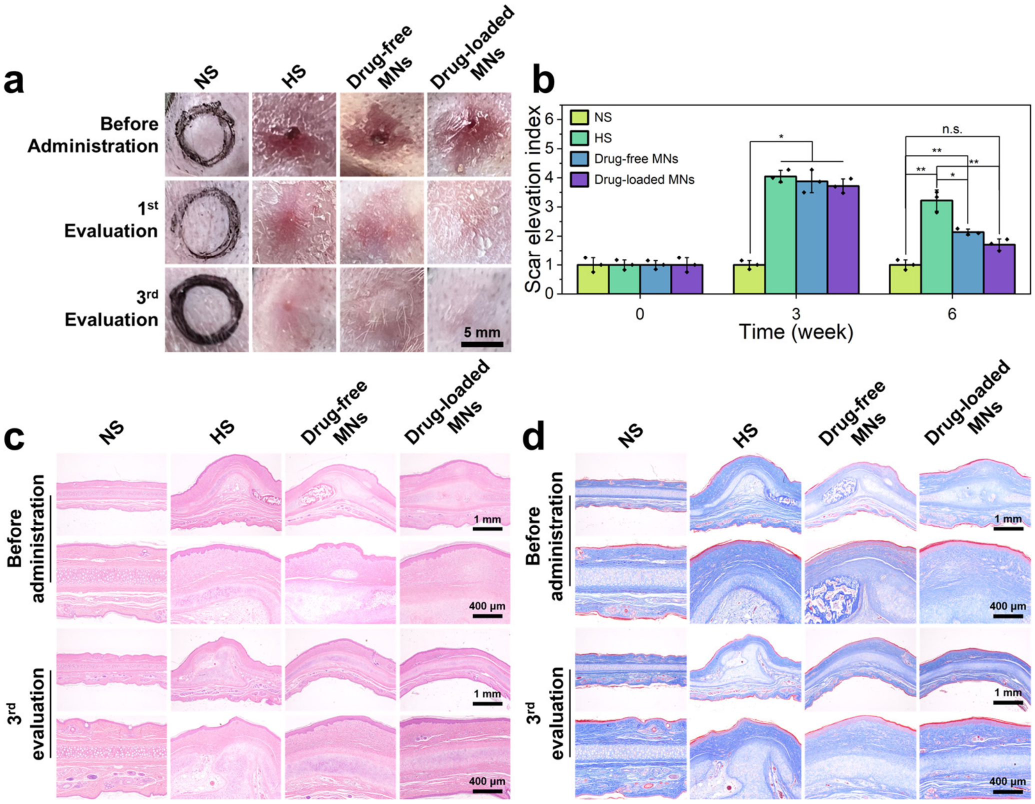

Supplementary Fig. 17 Evaluation of in vivo therapeutic effect on HS with drug-loaded MNs patch after long-term storage. a, Representative photographs of HS before and after different treatments. **b,** SEI of different groups after the 3rd evaluation. **c,** H&E staining of HS with different treatments before and after the 3rd evaluation. **d,** Masson's trichrome staining of HS with different treatments before and after the 3rd evaluation. The scale bar in the last image can be applied to the others in the same panel. Representative images from n = 3 biologically independent samples. The data are presented as mean ± SD (n = 3 biologically independent samples). The data were analyzed using one-way ANOVA. n.s. means no significance, *P < 0.05.

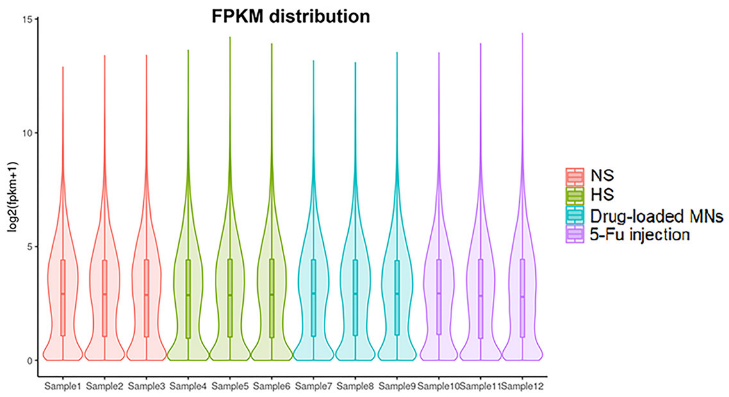

Supplementary Fig. 18 Distribution and correlation of gene expression. Violin diagram of the gene expression distribution by FPKM (Fragments per kilo base per million mapped reads) method across 12 samples (4 groups, n=3 biologically independent samples). The lower bound of box represents the first quartile (Q1; the 25th percentile) of the data, the line in the center of the box represents the median (Q2; the 50th percentile) of the data, the higher side represents the third quartile (Q3; the 75th percentile) of the data, the lower whisker shows the minima and its variability, and the higher whisker shows maxima and its variability. The width of each curve corresponds with the frequency of data points in each region.

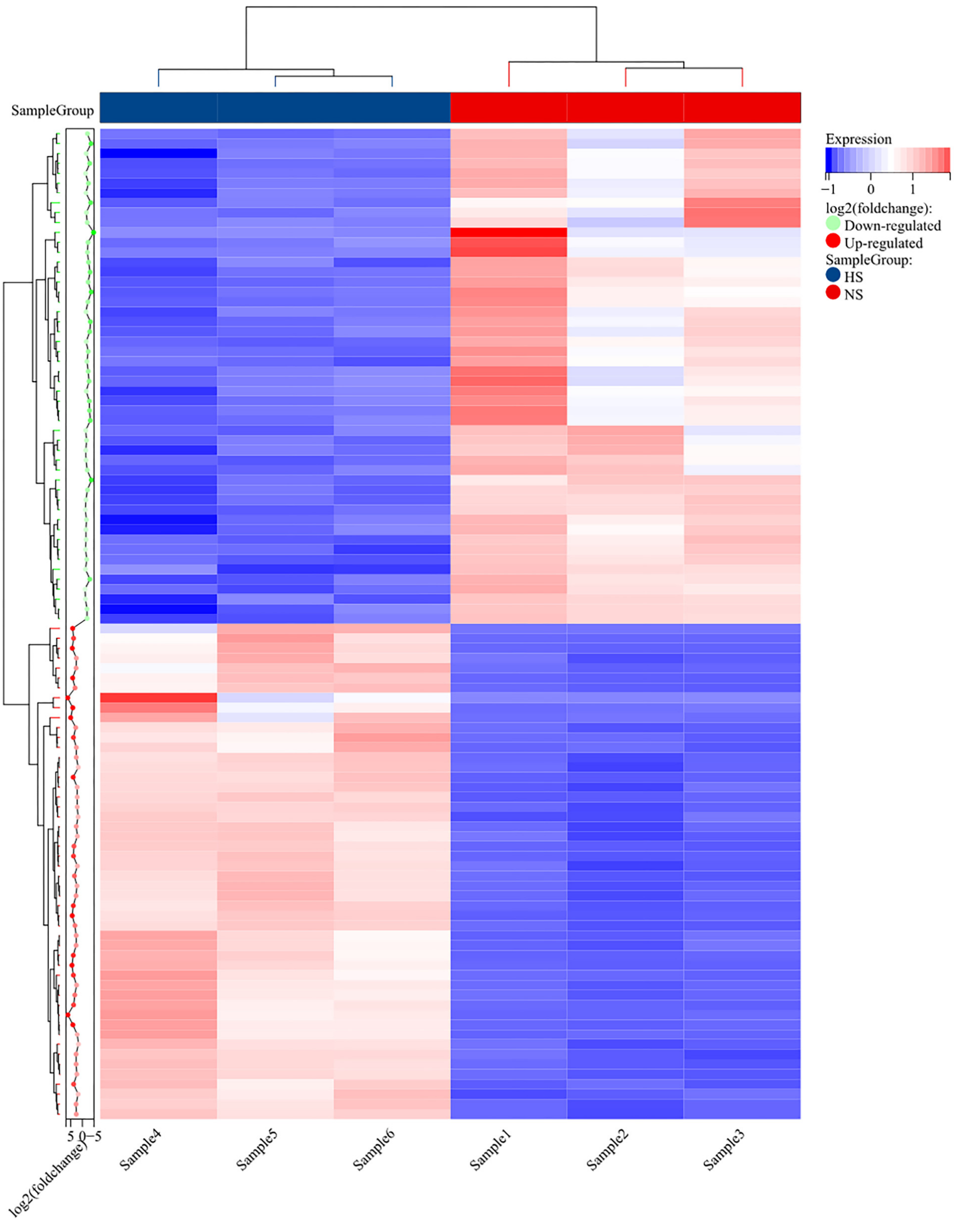

Supplementary Fig. 19 Identification of DEGs. Heatmap of DEGs between HS and NS groups.

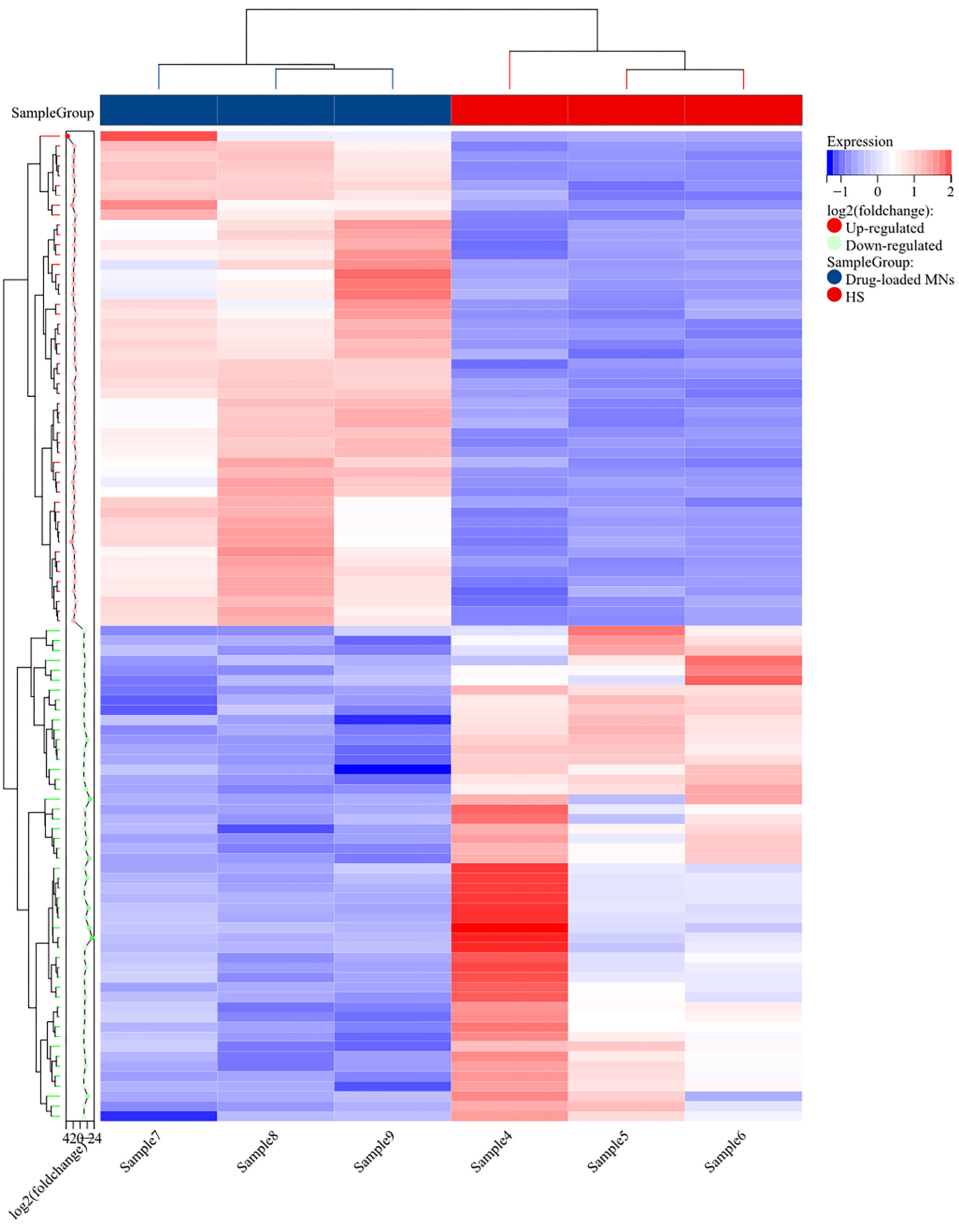

Supplementary Fig. 20 Identification of DEGs. Heatmap of DEGs between Drug-loaded MNs and HS groups.

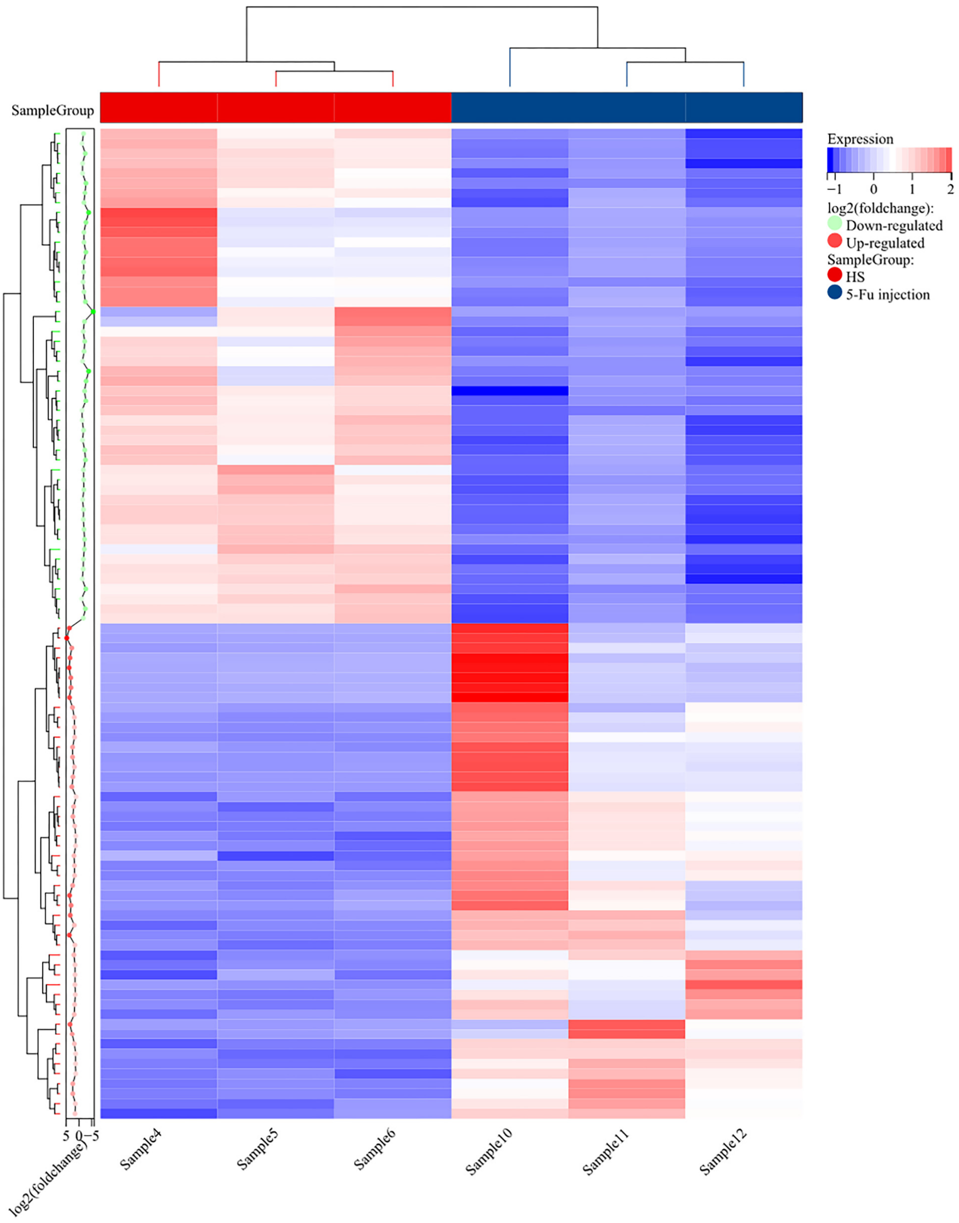

Supplementary Fig. 21 Identification of DEGs. Heatmap of DEGs identified between 5-Fu injection and HS groups.

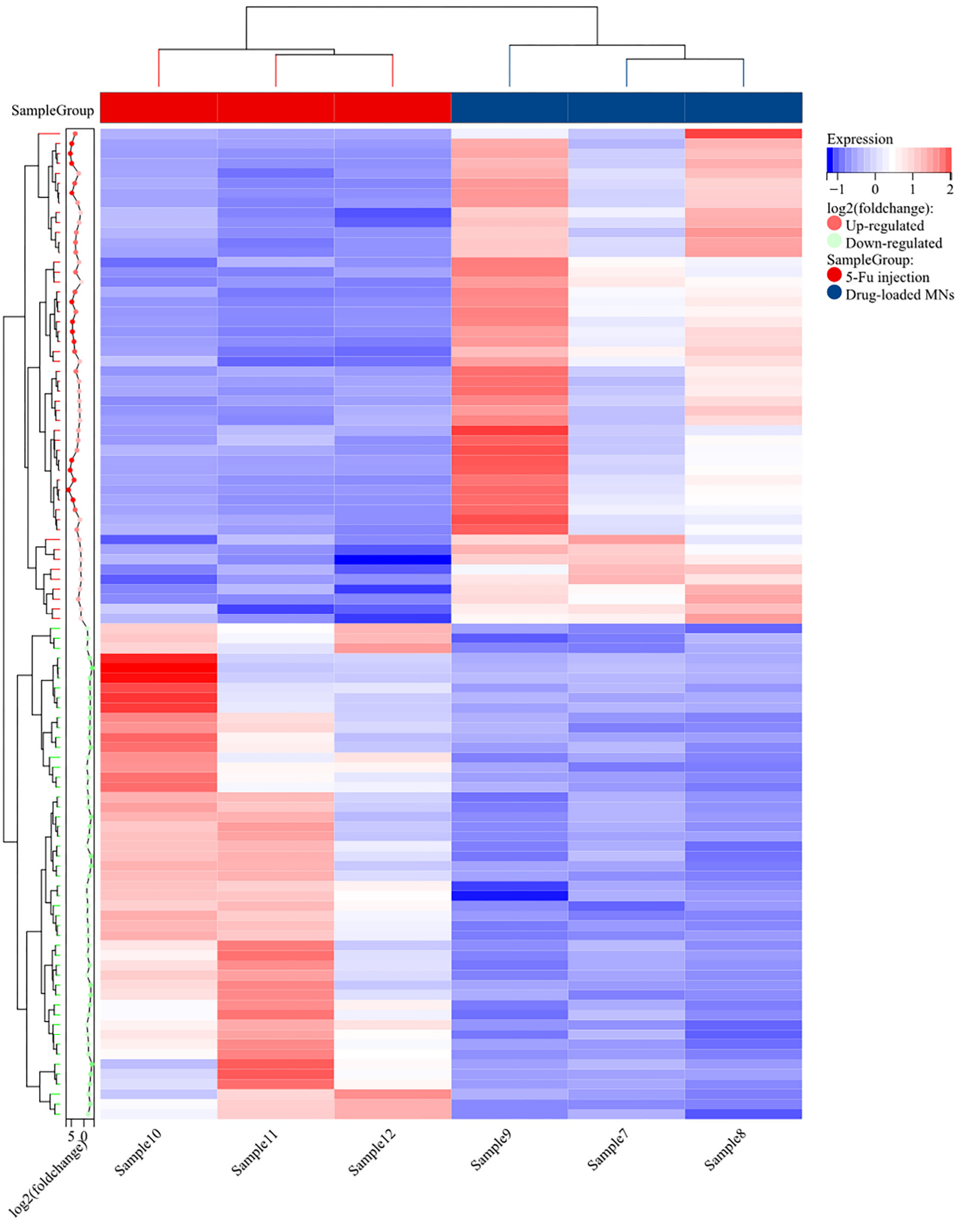

Supplementary Fig. 22 Identification of DEGs. Heatmap for DEGs identified between Dug-loaded MNs and 5-Fu injection groups.

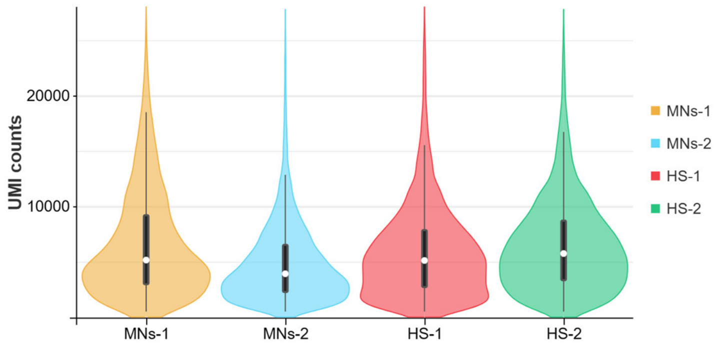

Supplementary Fig. 23 Quality control of study samples. Violin plots showed UMI counts of samples from 4 samples (2 groups, n=2 biologically independent samples). The lower bound of box represents the first quartile (Q1; the 25th percentile) of the data, the line in the center of the box represents the median (Q2; the 50th percentile) of the data, the higher side represents the third quartile (Q3; the 75th percentile) of the data, the lower whisker shows the minima and its variability, and the higher whisker shows maxima and its variability. The width of each curve corresponds with the frequency of data points in each region.

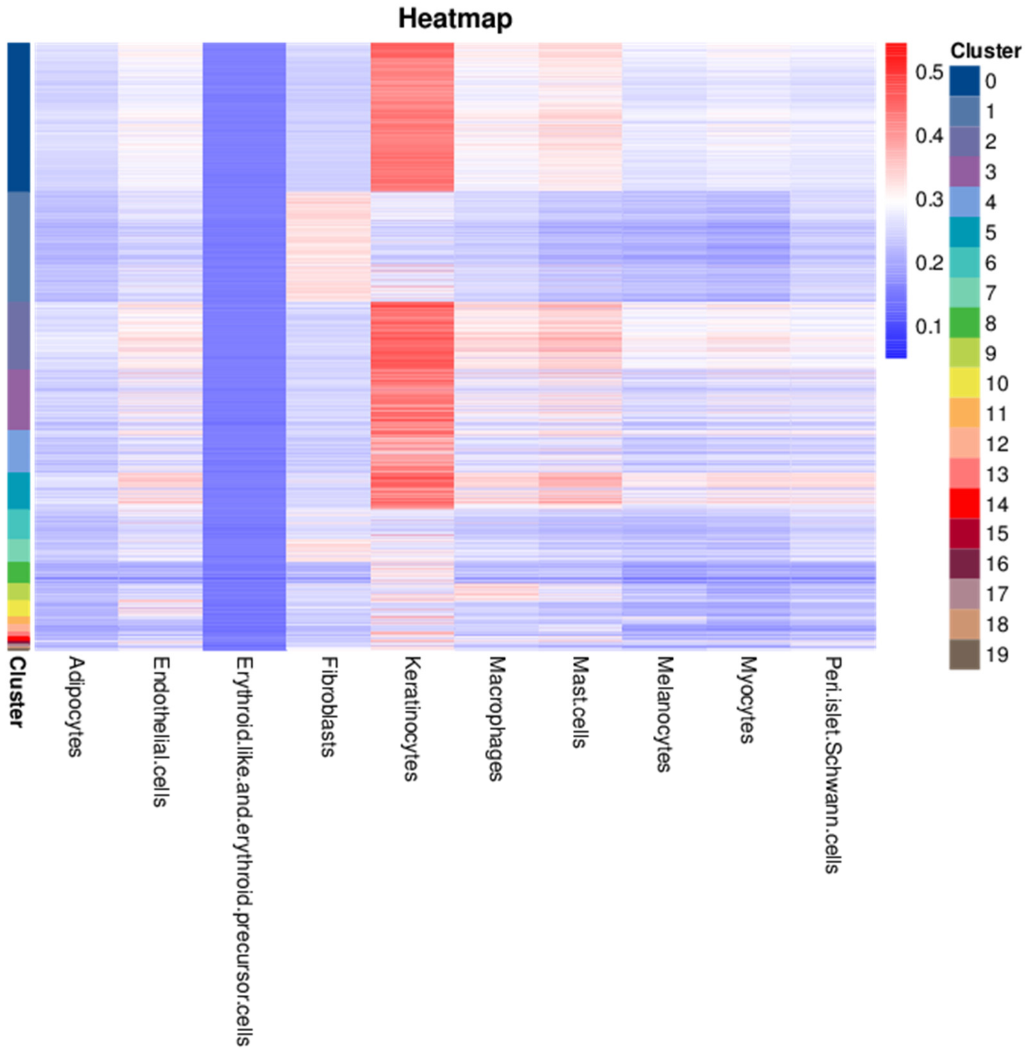

Supplementary Fig. 24 Cell type annotation. The heatmap shows the annotated cell type of each cell cluster.

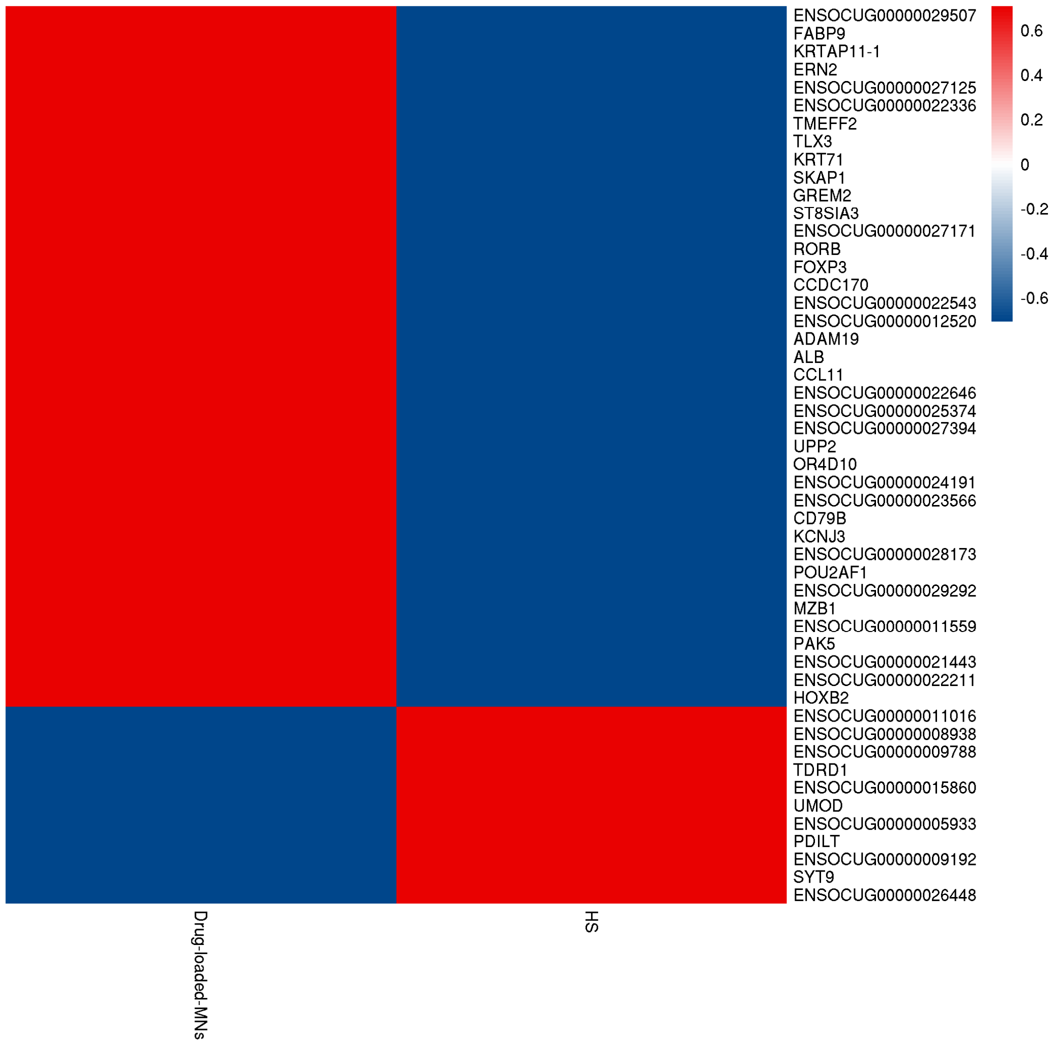

Supplementary Fig. 25 DEGs in keratinocytes. The heatmap shows the top 50 DEGs in keratinocytes between drug-loaded MNs and HS groups.

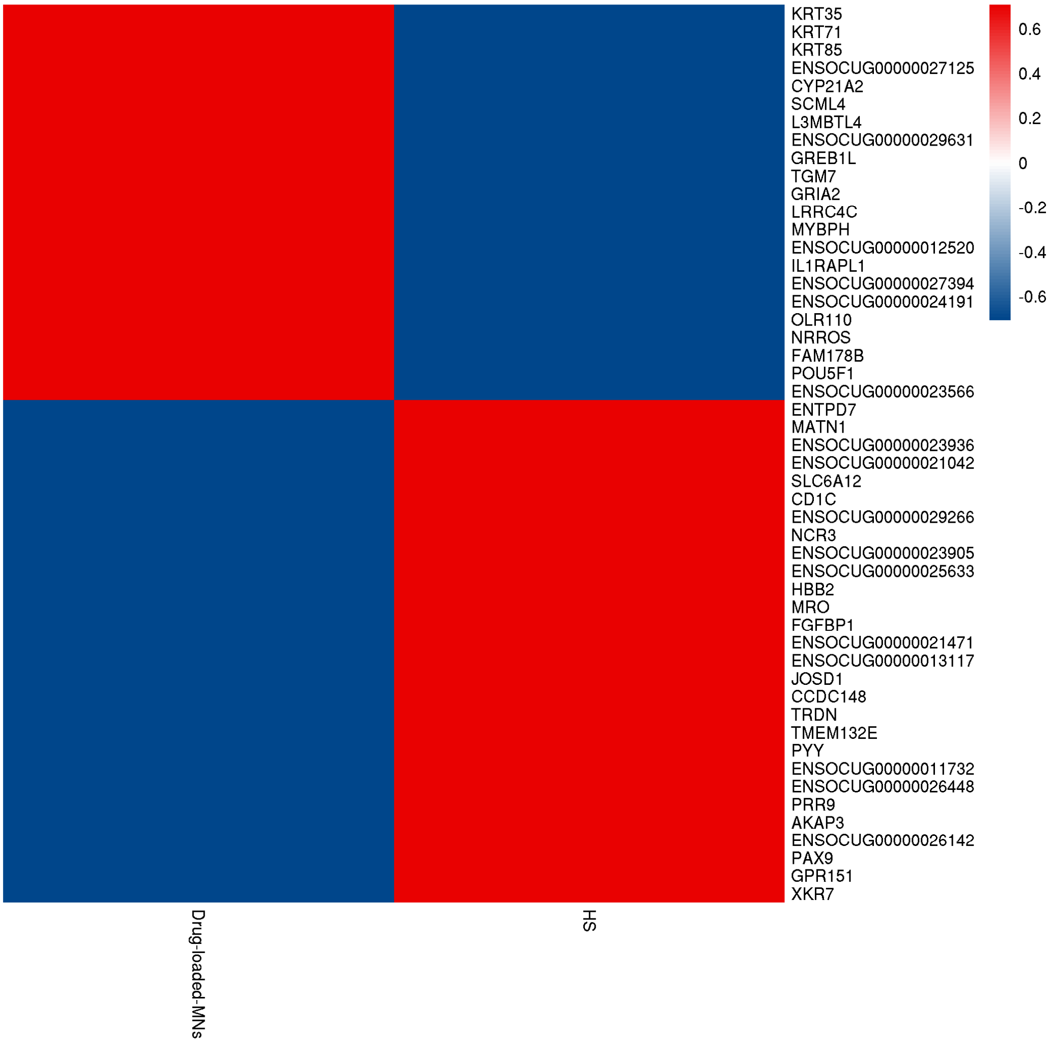

Supplementary Fig. 26 DEGs in fibroblasts. The heatmap shows the top 50 DEGs in fibroblasts between drug-loaded MNs and HS groups.

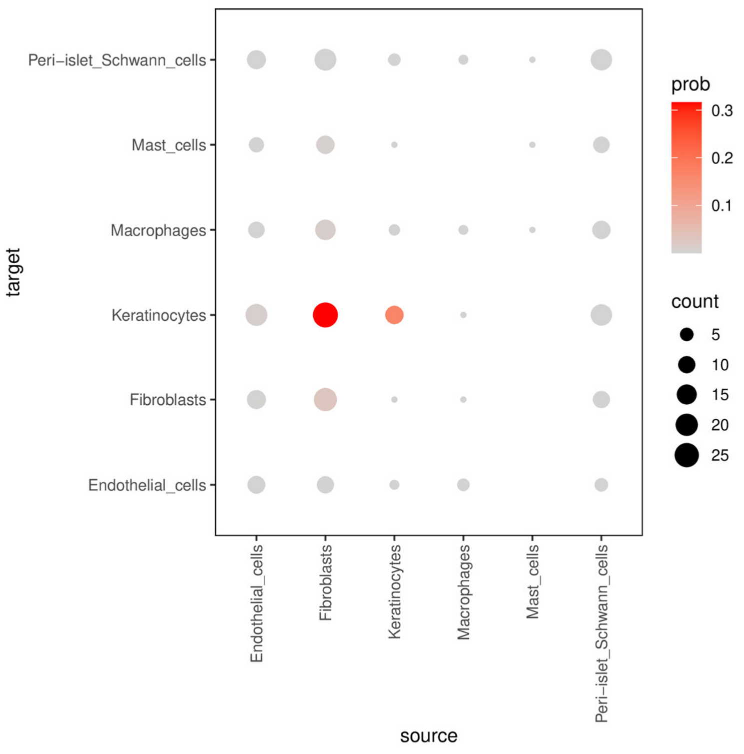

Supplementary Fig. 27 Intercellular interactions. Bubble plot of ligand-receptor relationships between different cell types. The size and color of bubble indicates the number of ligand and receptor pairs and the probability of communication between the cell types.

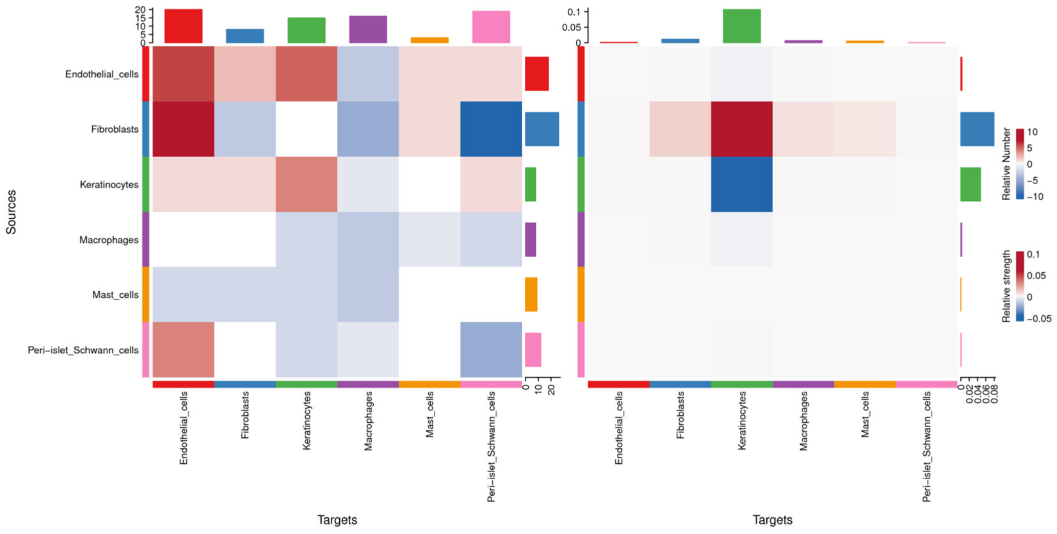

Supplementary Fig. 28 Heat map of intercellular communication. The left figure depicts the difference in the number of intercellular ligand-receptor pairs, while the right figure shows the difference in the strength of intercellular ligand-receptor pairs.

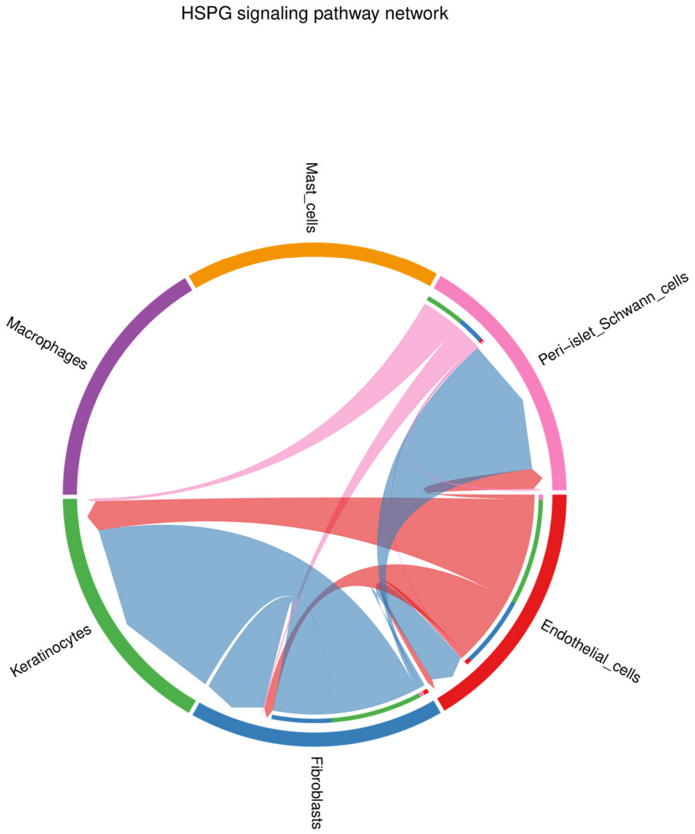

Supplementary Fig. 29 Circos plot of incellular communication. The circos plot depicts the expression level of HSPG2-DAG1 signaling pathway between different cell types.

Supplementary Tables

Supplementary Table 1 The top 20 hub genes ranked by 6 algorithms in cytoHubba.

MCC	MNC	Degree	EPC	Radiality	Stress
FLG2	*DSG1*	*FLG2*	*DSC1*	*JUP*	*IGF1*
DSG1	*FLG2*	*DSG1*	*DSG1*	*FLG2*	*MST1R*
EVPL	*DSC1*	*DSC1*	*FLG2*	*SPINK5*	*CDSN*
KRT10	*EVPL*	*CDSN*	*EVPL*	*DSC1*	*FLG2*
CDSN	*CDSN*	*EVPL*	*KRT10*	*DSG1*	*JUP*
KRT1	*SPINK5*	*SPINK5*	*CDSN*	*PKP1*	*IL36RN*
DSP	*KRT10*	*PKP1*	*SPINK5*	*EVPL*	*SPINK5*
DSC1	*KRT1*	*KRT10*	*DSP*	*ABCA12*	*DSC1*
SPINK5	*PKP1*	*KRT1*	*PKP1*	*DSC3*	*DSG1*
DSC3	*PNPLA1*	*JUP*	*KRT1*	*DSP*	*PNPLA1*
PKP1	*DSP*	*DSP*	*NIPAL4*	*IL36RN*	*NES*
ABCA12	*ABCA12*	*PNPLA1*	*ABCA12*	*IGF1*	*ABCA12*
JUP	*KRT5*	*MST1R*	*PNPLA1*	*NIPAL4*	*FOS*
PNPLA1	*NIPAL4*	*ABCA12*	*DSC3*	*PNPLA1*	*MIXL1*
ALOX12B	*JUP*	*NIPAL4*	*JUP*	*CDSN*	*KRT10*
NIPAL4	*CERS3*	*KRT5*	*KRT5*	*KRT5*	*EVPL*
KRTDAP	*DSC3*	*CERS3*	*ALOX12B*	*KRT10*	*COL17A1*
CERS3	*KRTDAP*	*DSC3*	*KRTDAP*	*KRT1*	*PKP1*
PERP	*TGM3*	*FOS*	*CERS3*	*MST1R*	*NIPAL4*
TGM3	*ALOX12B*	*KRTDAP*	*TGM3*	*TGM5*	*SULT2B1B*

Supplementary References

1. Zhu, J. et al. Gelatin methacryloyl microneedle patches for minimally invasive extraction of skin interstitial fluid. *Small* **16**, 1905910 (2020).
2. Ovsianikov, A. et al. Laser fabrication of three-dimensional CAD scaffolds from photosensitive gelatin for applications in tissue engineering. *Biomacromolecules* **12**, 851-858 (2011).
3. Chang, H. et al. A swellable microneedle patch to rapidly extract skin interstitial fluid for timely metabolic analysis. *Adv. Mater.* **29**, 1702243 (2017).
