# Supplementary material for: Endogenous stimuli-responsive separating microneedles to inhibit hypertrophic scar through remodeling the pathological microenvironment
Source: Nat Commun. 2024 Mar 6;15:2038. doi: 10.1038/s41467-024-46328-2 (PMC10917775; doi:10.1038/s41467-024-46328-2)
Supplement: Supplementary file 2 — Reporting Summary [file 41467_2024_46328_MOESM2_ESM.pdf]

nature portfolio

Corresponding author(s): Jintao Zhu

Last updated by author(s): Feb 3, 2024

Reporting Summary

Nature Portfolio wishes to improve the reproducibility of the work that we publish. This form provides structure for consistency and transparency in reporting. For further information on Nature Portfolio policies, see our Editorial Policies and the Editorial Policy Checklist.

Statistics

For all statistical analyses, confirm that the following items are present in the figure legend, table legend, main text, or Methods section.

n/a	Confirmed	
☐	☒	The exact sample size (*n*) for each experimental group/condition, given as a discrete number and unit of measurement
☐	☒	A statement on whether measurements were taken from distinct samples or whether the same sample was measured repeatedly
☐	☒	The statistical test(s) used AND whether they are one- or two-sided<br>*Only common tests should be described solely by name; describe more complex techniques in the Methods section.*
☒	☐	A description of all covariates tested
☐	☒	A description of any assumptions or corrections, such as tests of normality and adjustment for multiple comparisons
☐	☒	A full description of the statistical parameters including central tendency (e.g. means) or other basic estimates (e.g. regression coefficient) AND variation (e.g. standard deviation) or associated estimates of uncertainty (e.g. confidence intervals)
☐	☒	For null hypothesis testing, the test statistic (e.g. *F*, *t*, *r*) with confidence intervals, effect sizes, degrees of freedom and *P* value noted<br>*Give P values as exact values whenever suitable.*
☒	☐	For Bayesian analysis, information on the choice of priors and Markov chain Monte Carlo settings
☒	☐	For hierarchical and complex designs, identification of the appropriate level for tests and full reporting of outcomes
☒	☐	Estimates of effect sizes (e.g. Cohen's *d*, Pearson's *r*), indicating how they were calculated

Our web collection on statistics for biologists contains articles on many of the points above.

Software and code

Policy information about availability of computer code

Data collection	Bulk RNA sequencing was completed with an Illumina NovaSeq 6000 System (Illumina Inc., San Diego, CA). For single cell RNA sequencing, the Single Cell 3' Protocol produced Illumina-ready sequencing libraries. 10X Genomics Cell Ranger software (version 3.1.0) was used to convert raw BCL files to FASTQ files, alignment and counts quantification.
Data analysis	Data of RNA sequencing and single cell RNA sequencing were analyzed using R package of DESeq2 , DAVID Bioinformatics Resources (Version 2021), GSEA analysis tool (Version 4.1.1), STRING database (Version 11.5), CellPhoneDBv2 (doi:10.1038/s41596-020-0292-x), and CellChat (doi: 10.1038/s41467-021-21246-9). Image J software (Java-8 versions) was used to calculate wound healing rate of in vitro wound scratch assay and scar elevation index of in vivo histological analysis. Statistical analysis was performed by IBM SPSS Statistics 25.

For manuscripts utilizing custom algorithms or software that are central to the research but not yet described in published literature, software must be made available to editors and reviewers. We strongly encourage code deposition in a community repository (e.g. GitHub). See the Nature Portfolio guidelines for submitting code & software for further information.

Data

Policy information about availability of data

All manuscripts must include a data availability statement. This statement should provide the following information, where applicable:

- Accession codes, unique identifiers, or web links for publicly available datasets
- A description of any restrictions on data availability
- For clinical datasets or third party data, please ensure that the statement adheres to our policy

The authors declare that all the data supporting the findings of this study within the paper and the Supplementary Information are available from the corresponding author upon request. The bulk RNA sequencing data have been submitted to the NCBI Gene Expression Omnibus (GEO) datasets with accession number GSE254263. The single cell RNA sequencing data have been submitted to the NCBI GEO datasets with accession number GSE254543.

Research involving human participants, their data, or biological material

Policy information about studies with human participants or human data. See also policy information about sex, gender (identity/presentation), and sexual orientation and race, ethnicity and racism.

Reporting on sex and gender	The study explored the safety of soluble gelatin microneedles as a drug carrier system to human skin. Th sex and gender isn't a research variable in this study, thus sex and gender isn't applicable.
Reporting on race, ethnicity, or other socially relevant groupings	The study explored the safety of soluble gelatin microneedles as a drug carrier system to human skin, The race, ethnicity, or other socially relevant groupings were not research variables in this study, thus the race, ethnicity, or other socially relevant groupings were not applicable.
Population characteristics	The population characteristics included: 1)Adult male or female over 18 years; 2) Currently and past have no skin diseases and underlying diseases; 3) Not taken any antihistamines, vasodilators or constrictors, anticoagulants, hormones and immunosuppressants in the past 3 months.
Recruitment	The human subjects were recruited from outpatient patients at dermatology department of Wuhan Union Hospital, HUST through posters and website advertisement. Then two independent dermatologists selected the study subjects according to the inclusion and exclusion criteria to exclude potential self-selection bias.
Ethics oversight	The study protocol was approved by the Institutional Review Board at Wuhan Union Hospital, Tongji Medical College, HUST (Approval No. [2022] IACUC Number: 0509), and was carried out in accordance with the Declaration of Helsinki.

Note that full information on the approval of the study protocol must also be provided in the manuscript.

Field-specific reporting

Please select the one below that is the best fit for your research. If you are not sure, read the appropriate sections before making your selection.

☒ Life sciences ☐ Behavioural & social sciences ☐ Ecological, evolutionary & environmental sciences

For a reference copy of the document with all sections, see nature.com/documents/nr-reporting-summary-flat.pdf

Life sciences study design

All studies must disclose on these points even when the disclosure is negative.

Sample size	No statistical methods were used to predetermine sample sizes. Sample sizes were based on pilot experiments conducted in the same laboratory and comparable to similar studies in the field. The precise number of human subjects and animals were indicated in the figure legend.
Data exclusions	No data were excluded from the analyses.
Replication	All experiments were successfully replicated at least in 3 independent experiments.
Randomization	The in vivo skin irritation assay was self-control study to explore the skin irritation of the separating gelatin MNs used in human. 5 healthy human volunteers participated in the study, and measurements were performed on the treated skin before and at different time intervals after application of separating gelatin MNs. The rabbits of animal experiment were allocated in different groups randomly based on random number table.
Blinding	The evaluation and scoring of histopathology of HE-stained tissue sections was performed in a blinded fashion. In the other experiments, no blinding was used during allocation of experimental groups, because all data collection and analysis is quantitative and not qualitative in nature. To avoid introducing bias, samples were measured in a standardized way.

Reporting for specific materials, systems and methods

We require information from authors about some types of materials, experimental systems and methods used in many studies. Here, indicate whether each material, system or method listed is relevant to your study. If you are not sure if a list item applies to your research, read the appropriate section before selecting a response.

Materials & experimental systems

n/a	Involved in the study	
☐	☒	Antibodies
☐	☒	Eukaryotic cell lines
☒	☐	Palaeontology and archaeology
☐	☒	Animals and other organisms
☐	☒	Clinical data
☒	☐	Dual use research of concern
☒	☐	Plants

Methods

n/a	Involved in the study	
☒	☐	ChIP-seq
☒	☐	Flow cytometry
☒	☐	MRI-based neuroimaging

Antibodies

Antibodies used	Mouse monoclonal [6E3F8] to MMP2, Abcam, catalog # ab86607.<br>Mouse monoclonal [56-2A4] to MMP9, Abcam, catalog # ab58803.<br>Goat Anti-Mouse IgG H&L (HRP), Abcam, catalog # ab205719.<br>Donkey anti-Mouse IgG (H+L) Highly Cross-Adsorbed Secondary Antibody, Alexa Fluor 594, Thermo Fisher Scientific, catalog # A-21203.<br>8-OHdG Antibody (E-8), Santa Cruz Biotechnology, catalog # sc-393871
Validation	Mouse monoclonal [6E3F8] to MMP2, Abcam, catalog # ab86607:<br>https://www.abcam.cn/products/primary-antibodies/mmp2-antibody-6e3f8-ab86607.html<br>Mouse monoclonal [56-2A4] to MMP9, Abcam, catalog # ab58803:<br>https://www.abcam.cn/products/primary-antibodies/mmp9-antibody-56-2a4-ab58803.html<br>Goat Anti-Mouse IgG H&L (HRP), Abcam, catalog # ab205719:<br>https://www.abcam.cn/products/secondary-antibodies/goat-mouse-igg-hl-hrp-ab205719.html<br>Donkey anti-Mouse IgG (H+L) Highly Cross-Adsorbed Secondary Antibody, Alexa Fluor 594, Thermo Fisher Scientific, catalog # A-21203:<br>https://www.thermofisher.cn/cn/zh/home.html?<br>CID=ebz_bus_sbu_r04_cn_0se_bdk_pt_pur_Life_gene_aBrand_pc_mkt_050821_0000000A84856FE1<br>8-OHdG Antibody (E-8), Santa Cruz Biotechnology, catalog # sc-393871:<br>https://www.scbt.com/zh/p/8-ohdg-antibody-e-8?requestFrom=search

Eukaryotic cell lines

Policy information about cell lines and Sex and Gender in Research

Cell line source(s)	Standard fibroblast cell line (NIH/3T3) was supplied by the Cell Bank of the Chinese Academy of Sciences (Shanghai, China). The human hypertrophic scar fibroblast (HSFb) was obtained from Hubei Engineering Research Center for Skin Repair and Theranostics (Wuhan, China).
Authentication	HSFb cells were identified by vimentin immunofluorescence and the purity was over 90%. NIH/3T3 cells were identified by Short Tandem Repeat (STR).
Mycoplasma contamination	All cell lines tested negative for mycoplasma contamination.
Commonly misidentified lines (See ICLAC register)	None of any commonly misidentified cell lines used in this study.

Animals and other research organisms

Policy information about studies involving animals; ARRIVE guidelines recommended for reporting animal research, and Sex and Gender in Research

Laboratory animals	Fifteen female rabbits (10-12 week, ~2.3-2.7 kg) were purchased from Experimental Animal Center of Tongji Medical College, Huazhong University of Science and Technology (HUST; Wuhan, China). All experimental animals were caged individually, fed at the temperature of 25±2 °C and relative humidity of 55%±2%, and maintained under standard conditions with a 12-h light/dark cycle. Female BALB/c mice (8-10 week, ~18-20 g) were purchased from Liaoning Changsheng Biotechnology Co, td.(Liaoning, China). The mice were kept at the temperature of 25±2 °C and relative humidity of 55%±2% under standard conditions. All the mice were fed ad libitum, and allowed free access to water.

Wild animals	This study didn't involve wild animals.
Reporting on sex	This study didn't involve reporting on sex.
Field-collected samples	The study didn't involve field-collected samples.
Ethics oversight	All the animal experiments were performed in line with the guidelines of "The Care and Use of Laboratory Animals in Huazhong University of Science and Technology", as well as approved by the Institutional Animal Care and Use Committee of Tongji Medical College, Huazhong University of Science and Technology (Approval No. [2021] IACUC Number: 2751) .

Note that full information on the approval of the study protocol must also be provided in the manuscript.

Clinical data

Policy information about clinical studies

All manuscripts should comply with the ICMJE guidelines for publication of clinical research and a completed CONSORT checklist must be included with all submissions.

Clinical trial registration	The study protocol was approved by the Institutional Review Board at Wuhan Union Hospital, Tongji Medical College, HUST (Approval No. [2022] IACUC Number: 0509).
Study protocol	The study protocol was available from Institutional Review Board at Wuhan Union Hospital, Tongji Medical College, HUST.
Data collection	The preclinical study was conducted in dermatology department of Wuhan Union Hospital, HUST from September 1, 2022 to December 31, 2022. Medical records, photos, and experimental results will be collected, recorded and analyzed by the Department of Dermatology of Wuhan Union Hospital. Data collection was performed by clinical researchers under the supervision of the principal, who will be responsible for the accuracy, completeness, and timeliness of the reported data. All data shall be clear to ensure its traceability. The personal data, photos and test results of the subjects in the trial was kept strictly confidential, only available to the relevant researchers of this project.
Outcomes	Main study endpoints/outcomes: transdermal water loss rate (TEWL), skin melanin and heme (Mexameter), skin epidermal moisture (Corneometer), and skin elasticity test (Reviscometer) after microneedle treatment. Secondary end points: erythema and edema of the inner forearm after the application of microneedles.
